# Genetic analysis of circulating metabolic traits in 619,372 individuals

**DOI:** 10.1038/s41586-026-10532-5

**Published:** 2026-05-20

**Authors:** Ralf Tambets, Mihkel Jesse, Jaanika Kronberg, Adriaan van der Graaf, Erik Abner, Urmo Võsa, Ida Rahu, Nele Taba, Anastassia Kolde, Dzvenymyra Yarish, Sariyya Abdullayeva, Anastasiia Alekseienko, Andres Veidenberg, Estonian Biobank Research Team, Mari Nelis, Georgi Hudjasov, Mait Metspalu, Reedik Mägi, Andres Metspalu, Lili Milani, Krista Fischer, Zoltán Kutalik, Tõnu Esko, Kaur Alasoo, Priit Palta

**Affiliations:** 1 Institute of Computer Science, University of Tartu, Tartu, Estonia; 2 Estonian Genome Centre, Institute of Genomics, University of Tartu, Tartu, Estonia; 3 Institute of Mathematics and Statistics, University of Tartu, Tartu, Estonia; 4 Department of Computational Biology, University of Lausanne, Lausanne, Switzerland; 5 University Center for Primary Care and Public Health, Unisanté, University of Lausanne, Lausanne, Switzerland

**Keywords:** Genome-wide association studies, Risk factors, Quantitative trait loci, Metabolomics

## Abstract

Interpreting the association of genetic variants with complex traits can be improved by gaining a greater understanding of the molecular consequences of these variants. Although genome-wide association studies (GWAS) for complex diseases routinely profile over one million individuals^1–5^, studies of molecular traits have lagged behind. Here we performed a GWAS meta-analysis for 249 circulating metabolic traits in the Estonian Biobank and the UK Biobank in up to 619,372 individuals. We identified 88,127 common and low-frequency locus–trait associations from 8,398 loci that converged on shared genes and pathways. Using statistical fine mapping, systematic phenome-wide colocalization and *cis*-Mendelian randomization, we explored putative causal links between metabolic traits and disease outcomes. We predict that although plasma branched-chain amino acids (BCAAs) have been associated with type 2 diabetes in observational studies^6,7^, lowering BCAA levels by targeting the BCAA catabolism pathway is unlikely to reduce type 2 diabetes risk. Leveraging our large sample size and high-quality genotype imputation, we found that 19.4% of the confidently fine-mapped variants had minor allele frequencies between 0.1 and 1%, and these variants were twofold enriched for predicted missense and splice-altering variants. Our results highlight the value of integrating low-frequency variants into genetic association studies.

## Abstract (web summary)

A genome-wide association study combining data from the Estonian Biobank and the UK Biobank identifies many common and low-frequency locus–metabolic trait associations, enabling the identification of putative causal links with disease outcomes.

## Main

Recent large-scale GWAS of metabolic traits have continued to uncover novel associations and biological insights^8–14^. However, for more than half of the metabolic traits that are captured by nuclear magnetic resonance (NMR) spectroscopy, the proportion of heritability explained by genome-wide significant variants remains below 50% (ref. ^12^), indicating that much larger sample sizes are needed to identify the remaining genetic effects. Furthermore, most existing association studies using the Nightingale Health NMR platform have been limited to common variants^8–10,12^ and exome sequencing^13,15^, leaving the full genome-wide spectrum of low-frequency genetic variation unexplored. Finally, larger sample sizes and increased statistical power also bring new challenges for interpreting genetic associations, particularly when genetic variants have pleiotropic effects on several correlated metabolic traits^8–10^. In particular, there is a growing concern that naive use of these associations in the Mendelian randomization^16^ framework can lead to spurious and misleading findings^17,18^.

## Association testing and meta-analysis

**Extended Data Fig. 1:**
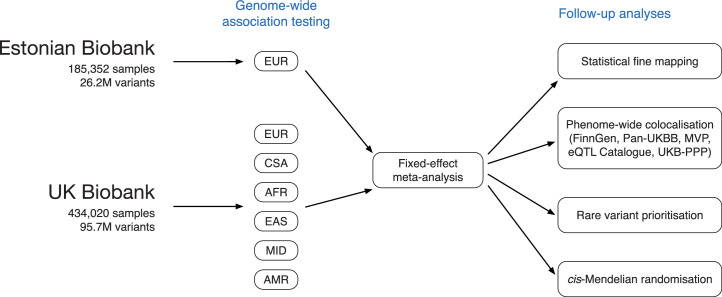
Outline of the study. First, a separate GWAS was performed for each metabolic trait in the Estonian Biobank and six genetic ancestry groups of the UK Biobank: EUR (European), AFR (African), AMR (Admixed American), MID (Middle Eastern), EAS (East Asian), CSA (Central/South Asian) as defined by Pan-UKBB. These ancestry-specific GWAS results were then combined in a fixed-effect meta-analysis. Finally, several follow-up analyses were performed to demonstrate the value of the resource. MVP - Million Veterans Program; UKB-PPP - UK Biobank Pharma Proteomics Project.

We performed GWAS for 249 metabolic traits (Supplementary Table 1) in the Estonian Biobank (EstBB; *n* = 185,352) and 6 genetic ancestry groups from the UK Biobank (UKBB; *n* = 434,020) (Extended Data Fig. 1). The UKBB genetic ancestry groups were defined previously by the Pan-UKBB project^19^ and are listed in Table 1. Relying on the population-specific genotype imputation panel for the EstBB^20^ and the Genomics England^21^ and TopMed^22^ imputation panels for the UKBB allowed us to test 10–96 million variants across genetic ancestry groups (up to nine times more than previous studies using the same NMR platform^8,12,13^). On the basis of minor allele frequency (MAF), we stratified these variants into three bins: common variants (MAF > 1%), low-frequency variants (MAF between 0.1% and 1%) and rare variants (MAF < 0.1%). The number of significant locus–trait pairs ranged from 37 (UKBB_AMR) to 62,543 (UKBB_EUR), and the number of independent lead variants (*r*^2^ < 0.8) ranged from 24 to 6,014, with most associations detected in the UKBB_EUR and EstBB subsets (Table 1). We observed high genetic correlation for matched metabolic traits between the EstBB (*n* = 185,352) and UKBB_EUR (*n* = 413,897) subsets (median genetic correlation (rg) = 0.91, mean rg = 0.89), indicating that genetic effects are largely shared between the two biobanks (Supplementary Table 2).

**Table 1:** Number of significant locus–metabolic trait pairs and unique lead variants (*r*^2^ < 0.8) detected in each genetic ancestry group and the two meta-analyses

Biobank and genetic ancestry group	Locus–trait pairs	Unique lead variants	Sample size
EstBB	26,736 (1,012)	2,313 (90)	185,352
UKBB_EUR (European)	62,543 (1,498)	6,014 (142)	413,897
UKBB_CSA (Central/South Asian)	1,070	113	8,652
UKBB_AFR (African)	916	143	6,439
UKBB_EAS (East Asian)	358	41	2,604
UKBB_MID (Middle Eastern)	92	44	1,500
UKBB_AMR (Admixed American)	37	24	928
Meta_EUR	86,886 (2,124)	8,260 (156)	599,249
Meta_ALL	88,127 (2,089)	8,398 (156)	619,372

The significance threshold was set to *P* < 5 × 10^−8^ for common and low-frequency variants (MAF > 0.1%) and to *P* < 6.25 × 10^−10^ for rare variants (MAF < 0.1%). The numbers of rare variant associations are shown in parentheses. A version of this table further accounting for the 249 metabolic traits tested is presented in Extended Data Table 1.

**Fig. 1:**
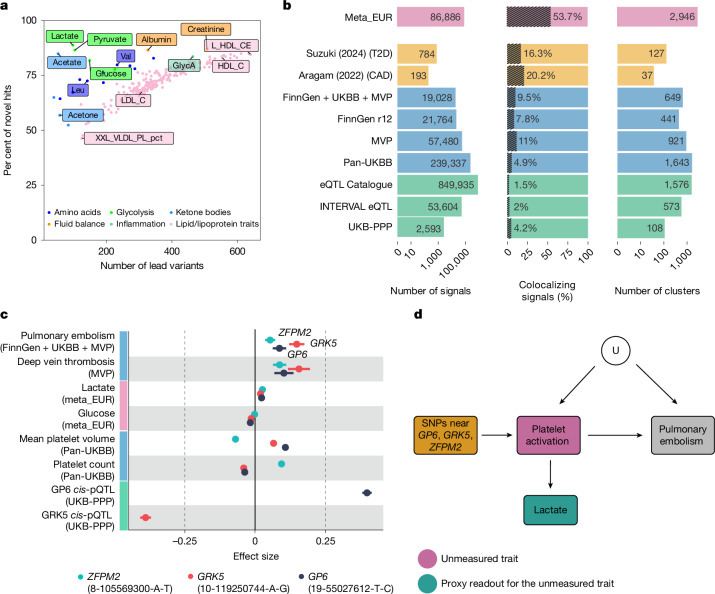
Known and novel genetic associations with metabolic traits. **a**, Number of genome-wide significant loci (*P* < 5 × 10^−8^) detected for each metabolic trait (*n* = 249) and the proportion of those associations that were not detected by Karjalainen et al.^8^. **b**, Overview of the datasets included in the colocalization analysis. The first column shows the number of signals included for colocalization from each dataset. The second column shows the proportion of these signals that colocalize with at least one metabolic trait. The third column indicates the number of colocalization clusters that involve at least one colocalization from each dataset. **c**, Forest plot of colocalizing genetic associations at the *ZFPM2*, *GRK5* and *GP6* loci between pulmonary embolism, deep vein thrombosis, lactate, glucose, mean platelet volume, platelet count and *cis*-pQTL signals for GP6 and GRK5 proteins. The points show the standardized GWAS effect size (beta) and error bars show the 95% confidence intervals. **d**, Proposed causal model linking genetic variants at the *GP6*, *GRK5* and *ZFPM2* loci via platelet activation to increased pulmonary embolism risk. Lactate is likely to act as a proxy readout of platelet activation without having a direct causal effect on pulmonary embolism risk. U, unmeasured confounders. Source data

In the meta-analysis of EstBB and UKBB_EUR (meta_EUR; *n* = 599,249), we identified 86,886 locus–trait pairs, corresponding to 8,260 independent lead variants (*r*^2^ < 0.8). This represented an approximately tenfold increase compared with Karjalainen et al.^8^ (*n* = 136,016; 8,578 locus–trait pairs) and a 63% increase compared with a parallel study by Zoodsma et al.^13^ on the overlapping set of UKBB samples (*n* = 450,016; 52,662 locus–trait pairs). The estimated heritability of individual metabolic traits ranged from 2.8% for acetoacetate to 19.5% for HDL_size (median 10.2%), and we observed a clear linear relationship between heritability and the number of loci associated with each metabolic trait (Supplementary Fig. 1 and Supplementary Table 3). On average, 93% of the lead variant associations detected by Karjalainen et al.^8^ were replicated in our meta_EUR analysis with a highly concordant direction of effect (Supplementary Fig. 2). We also detected many novel associations for all tested metabolic traits. The fraction of novel associations ranged from 27% for 3-hydroxybutyrate to 85% for lactate (Fig. 1a). Altogether, we identified 7,790 novel independent lead variants (*r*^2^ < 0.8) not previously reported by Karjalainen et al.^8^, including 163 lead variants on the X chromosome. To identify additional conditionally distinct association signals, we performed statistical fine mapping around the 84,762 meta_EUR locus–trait pairs that had MAF > 0.1% in the UKBB_EUR subset. We restricted the fine-mapping analysis to the summary statistics from the UKBB_EUR subset, as this allowed us to use in-sample linkage disequilibrium (LD) calculated from the overlapping set of 413,897 individuals and thus avoid a major potential source of false positives^23^. We identified 116,467 independent credible sets, 31,392 (27%) of which were fine-mapped to 3,000 distinct putative causal variants (posterior inclusion probability (PIP) > 0.8). These included 271 putative missense variants predicted by Ensembl VEP and 172 putative splice-altering variants predicted by either SpliceAI^24^ or AlphaGenome^25^ (Supplementary Table 4). Notably, 28 missense variants were also predicted to affect splicing. As an example, a fine-mapped missense variant (19-48806519-G-C, PIP = 1) in the *BCAT2* gene was associated with BCAA levels and was also predicted to disrupt splicing by SpliceAI^24^ (score = 0.41) and AlphaGenome^25^ (score = 0.143)^26^.

In addition to the EUR genetic ancestry group, we also performed GWAS in five smaller genetic ancestry groups from the UKBB (AFR, AMR, CSA, EAS and MID) (Table 1). Including these summary statistics in our meta-analysis (meta_ALL) increased the number of independent lead variants from 8,260 to 8,398 (Table 1), 43 of which were not tested in the EstBB and UKBB_EUR cohorts owing to low allele frequency (allele count <20). Focusing separately on each genetic ancestry group, we found that between 9 and 48% of the lead variants had MAF < 0.1% in the UKBB_EUR analysis, with the highest proportion observed in the UKBB_AFR subset (68 out of 143 lead variants) (Extended Data Table 2). This highlights the need to substantially increase the sample sizes for under-represented genetic ancestry groups to enable the discovery of ancestry-specific associations^27^.

## Colocalization across molecular layers

To demonstrate how genetic associations with metabolic traits can help to interpret disease associations, we used gpu-coloc^26^ to colocalize all 86,886 signals from our meta_EUR analysis with GWAS summary statistics for up to 7,228 traits across three biobanks (FinnGen r12^28^, Pan-UKBB^19^, Million Veterans Program (MVP)^29^ and FinnGen+MVP + UKBB meta-analysis (https://public-mvp-ukbb.finngen.fi/)) as well as gene expression, splicing and protein QTLs from the expression quantitative trait loci (eQTL) Catalogue^30^, the INTERVAL eQTL study^31^ and the UKBB Pharma Proteomics Project (UKB-PPP)^32^. We also included GWAS meta-analysis summary statistics for coronary artery disease (CAD; Aragam (2022))^5^ and for type 2 diabetes (T2D; Suzuki (2024))^1^ (Fig. 1b). Using a stringent colocalization posterior probability (CLPP) threshold ((PP.H4) > 0.9), we detected a total of 932,864 colocalization events which involved all 249 studied metabolic traits at least once. We detected at least one colocalization for 53.4% of the metabolic trait signals. Similarly, 20.2% of the CAD loci and 16.3% of the T2D loci colocalized with at least one metabolic trait. For biobanks, the percentage of colocalizing loci ranged from 4.9% (Pan-UKBB) to 11% (MVP). The lowest rate of colocalization was observed for eQTLs and protein quantitative trait loci (pQTLs), where 1.5–4.2% of the loci colocalized with at least one metabolic trait. Finally, approximately 2.5% of these colocalizations involved low-frequency (MAF < 1%) metabolic trait loci.

**Extended Data Fig. 2:**
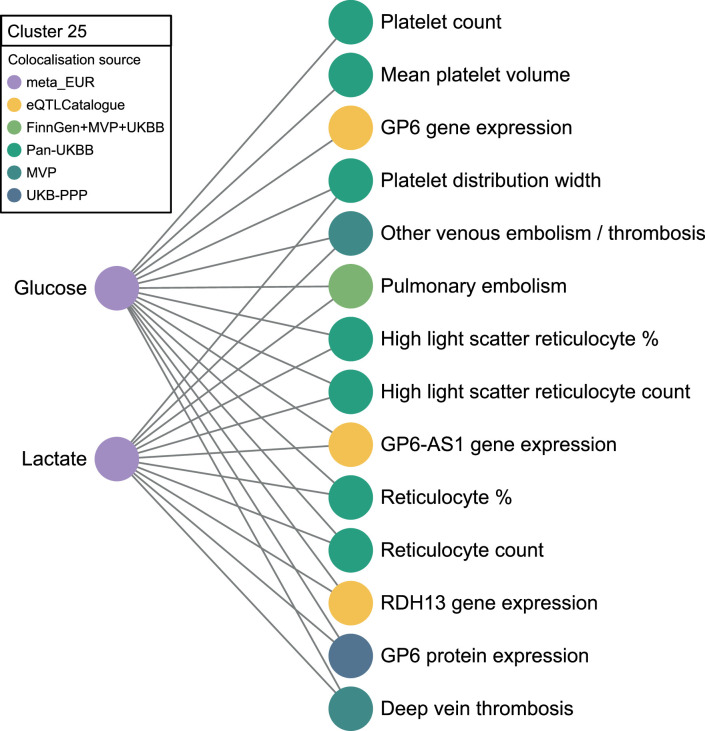
Colocalization graph at the *GP6* locus. Nodes correspond to association signals, and each edge indicates a colocalisation event (PP.H4 > 0.9) between the two signals. The graph has a bipartite structure, because we only tested for pairwise colocalizations between metabolic traits (left) and signals from other datasets (right). The genome-wide graph is sparse, making the identification of connected components (colocalisation clusters) straightforward.

To better understand the patterns of shared colocalizations across complex traits, metabolic traits and molecular QTLs, we converted the 932,864 colocalization events into a colocalization graph, where independent association signals were defined as nodes and colocalization events (PP.H4 > 0.9) between these nodes were defined as edges. This graph contained 2,946 connected components, which we defined as colocalization clusters (see example in Extended Data Fig. 2). The sizes of the clusters ranged from 1 edge to 62,504 edges and the number of metabolic traits in each cluster ranged from 1 to 228. The two largest sources of colocalizing associations were the Pan-UKBB (involved in 1,643 clusters) and the eQTL Catalogue (involved in 1,576 clusters), which is likely to reflect the very large number of associations present in those datasets (Fig. 1b). As expected, we re-discovered several known colocalizations involving CAD, T2D and inflammatory conditions (Supplementary Note 1).

## Plasma lactate and platelet activation

**Extended Data Fig. 3:**
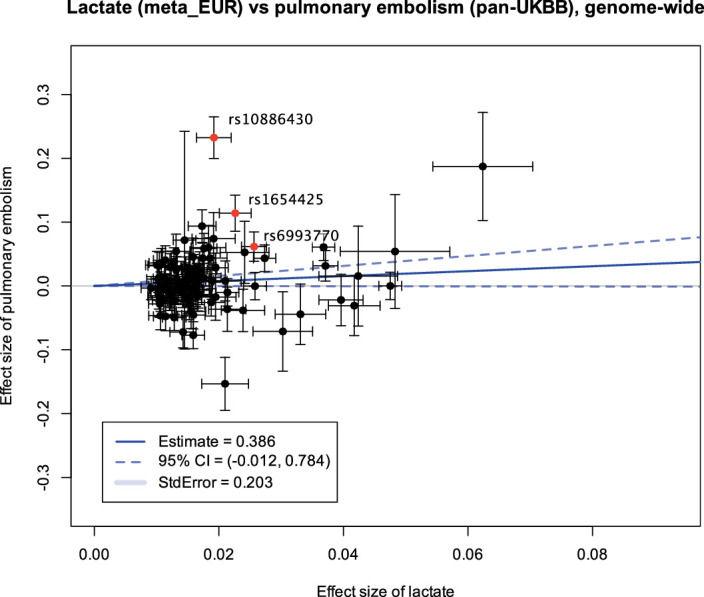
Genome-wide random-effect inverse variance weighted MR using lactate (meta_EUR) as exposure and pulmonary embolism (FinnGen + MVP + UKBB) as outcome. The points represent independent genome-wide significant (p < 5 × 10^−8^) GWAS hits for lactate in the meta_EUR meta-analysis. The lead variants at the *GP6* (rs1654425, 19-55027612-T-C), *GRK5* (rs10886430, 10-119250744-A-G) and *ZFPM2* (rs6993770, 8-105569300-A-T) loci have been highlighted. The points represent the standardized effect sizes of each genetic variant on the two traits with the error bars representing 95% confidence intervals. Source data

To prioritize novel disease colocalizations while limiting the effect of horizontal pleiotropy that could complicate interpretation, we focused on the 291 colocalization clusters that colocalized with at least one disease end-point from the FinnGen + UKBB + MVP meta-analysis and involved 5 or fewer metabolic traits. This analysis revealed three high-confidence colocalizations between pulmonary embolism and plasma lactate at the *GP6* (rs1654425, 19-55027612-T-C, cluster 25; Extended Data Fig. 2), *GRK5* (rs10886430, 10-119250744-A-G, cluster 104) and *ZFPM2* (rs6993770, 8-105569300-A-T, cluster 696) loci (Fig. 1c and Supplementary Table 5). At all three loci, increased plasma lactate was associated with an increased risk of pulmonary embolism (FinnGen + UKBB + MVP meta-analysis) and an increased risk of deep vein thrombosis (MVP). For the *GP6* and *GRK5* loci, we also detected colocalizations with the corresponding *cis*-pQTLs in the UKB-PPP plasma proteomics dataset, and for *GRK5* we also detected a colocalizing eQTL signal in platelets^33^ as well as in whole blood^31^. Although we did not detect a colocalizing *cis*-QTL effect at the *ZFPM2* locus, our lead variant (rs6993770) has previously been localized to a megakaryocyte-specific enhancer for other platelet traits^34^. Finally, although all three loci also colocalized with both platelet count and mean platelet volume, the effect size directions were not consistent. At the *GP6* and *GRK5* loci, increased mean platelet volume and decreased platelet count were associated with increased risk for pulmonary embolism, whereas at the *ZFPM2* locus, both effects were the opposite (Fig. 1c). To further characterize these three loci, we focused on fine-mapped credible sets from the *GP6*, *GRK5* and *ZFPM2* loci and performed colocalization against all harmonized GWAS credible sets from the Open Targets Platform^35^ using the CLPP method (Methods). We found that the *GP6* credible set (6 variants, maximum PIP = 0.48) colocalized (CLPP = 0.15) with a GWAS hit for platelet reactivity to collagen-related peptide^36^, first reported in ref. ^37^. Similarly, the fine-mapped variant at the *GRK5* locus (10-119250744-A-G, PIP = 0.99) colocalized (CLPP = 0.99) with a GWAS signal for thrombin-induced platelet activation in two independent studies^36,38^. This is consistent with experimental data in mice indicating that *GRK5* is a negative regulator of platelet activation^39^. The *ZFPM2* fine-mapped enhancer variant (8-105569300-A-T, PIP = 0.52) had pleiotropic effects on several blood cell type composition and cytokine traits^34^. Notably, at all three loci, the alleles associated with increased lactate and increased pulmonary embolism risk were also associated with a decrease in plasma glucose (Fig. 1c). As activated platelets generate energy by converting glucose to lactate^40,41^, this suggests that at these three loci, plasma lactate might serve as a biomarker for platelet activation^11^ (Fig. 1d). This mirrors observational studies linking higher plasma lactate levels to increased mortality in patients with pulmonary embolism^42–44^. However, our analysis does not imply that plasma lactate itself has a direct causal effect on pulmonary embolism risk. In fact, when we focused on all genome-wide variants associated with plasma lactate (including those not colocalizing with pulmonary embolism), the association was inconclusive at best (Extended Data Fig. 3).

## Low-frequency and rare variants

Whereas previous NMR GWAS studies have primarily focused on common variation (MAF > 1%)^8,12,13^, we tested all variants with minor allele count greater than 20. To further explore these low-frequency associations, we first focused on our fine-mapping results. We identified 10,016 (8.6%) credible sets where the MAF of the variant with the highest PIP was between 0.1% and 1%. These credible sets belonged to 786 probably independent signal clusters and contained 583 distinct confidently fine-mapped variants (PIP > 0.8). Notably, 135 out of 583 (23.1%) of the low-frequency fine-mapped variants were predicted to be missense or splice variants (Supplementary Table 4). By contrast, only 11.5% of the common (MAF > 1%) fine-mapped variants were predicted to alter coding sequence or splicing, highlighting the increased interpretability of many low-frequency associations. On the basis of this analysis, we prioritized several low-frequency missense variants in known metabolic genes, such as variants in *PCSK9*, *ABCA1* and *ANGPTL4* associated with lipid traits, a missense variant (12-102840493-G-A) in *PAH* associated with phenylalanine and tyrosine, a missense variant (X-24503382-A-G) in *PDK3* associated with pyruvate, and four independent missense variants in the *HAL* gene associated with histidine (Supplementary Table 4).

However, to ensure accurate LD information, our fine mapping was restricted to the UKBB_EUR subset of samples (69% of all samples) and only included variants with MAF > 0.1%. To find associations that might have been missed by fine mapping, we alternatively focused on all lead variants from the meta_EUR meta-analysis with MAF < 1% (*n* = 480 variants, 5.9% of all leads) (Supplementary Fig. 3). These corresponded to 324 low-frequency variants (MAF between 0.1% and 1%) and 156 rare variants with MAF < 0.1%. Reassuringly, 96 out of 324 low-frequency variants (30%) belonged to at least one fine-mapped credible set, and 68 out of 324 variants (21%) were predicted to be either missense or splice variants (Supplementary Table 6), suggesting that many of these low-frequency lead variants represent causal variants. The fraction of predicted missense and splice variants was slightly lower among the rare variants (23 out of 156 (15%)) (Supplementary Table 6), perhaps reflecting a slightly increased rate of false positives in this allele frequency bin or complex haplotype effects^45^.

## Convergence of common and rare variants

**Fig. 2:**
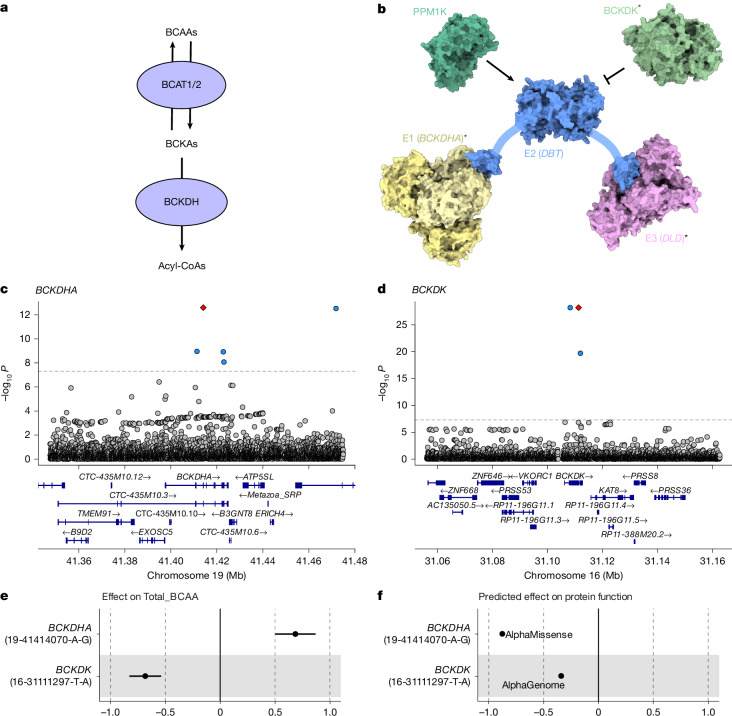
Convergence of common and low-frequency associations at the BCAA catabolism pathway. **a**, BCAAs are converted to branched-chain keto acids (BCKAs) by BCAA aminotransferase (encoded by *BCAT1* and *BCAT2*). This process is reversible. BCKAs can be further catabolised by the branched-chain α-keto acid dehydrogenase (BCKDH) complex to acyl-CoAs. **b**, The BCKDH complex is made up of subunits E1 (encoded by *BCKDHA* and *BCKDHB*), E2 (encoded by *DBT*) and E3 (encoded by *DLD*). The activity of the BCKDH complex is controlled by a kinase (BCKDK) that inhibits its function and a phosphatase (PPM1K) that reactivates it^46^. Asterisks indicate newly identified associations. **c**, GWAS association signal for total BCAA level (Total_BCAA) in the *BCKDHA* gene region. The *BCKDHA* missense variant 19-41414070-A-G is highlighted in red. **d**, GWAS association signal for Total_BCAA in the *BCKDK* gene region. The predicted *BCKDK* splice loss variant 16-31111297-T-A is highlighted in red. **e**, The effect of the *BCKDHA* missense variant and the *BCKDK* splice loss variant on Total_BCAA (beta ± 95% confidence interval). **f**, Predicted variant effect on protein function from AlphaMissense and AlphaGenome models (arbitrary units). Source data

As an example of insights gained from rare missense and splice variants, we observed convergence of common and rare variants on the BCAA catabolism pathway. The first two steps of BCAA catabolism are transamination of valine, leucine and isoleucine catalysed by branched-chain aminotransferase (encoded by *BCAT1* and *BCAT2* genes) followed by oxidative decarboxylation catalysed by the branched-chain α-keto acid dehydrogenase (BCKDH) complex^46^ (Fig. 2a). The BCKDH complex is made up of three proteins: the E1 subunit encoded by *BCKDHA* and* BCKDHB*, the E2 subunit encoded by *DBT*, and the E3 subunit encoded by *DLD*^46^ (Fig. 2b). The activity of the BCKDH complex is further regulated by BCKDK, which inhibits its activity, and protein phosphatase 2Cm (encoded by *PPM1K*), which reactivates it (Fig. 2b).

Common variant associations for three of the six genes (*BCAT2*, *DBT* and *PPM1K*) have been reported in previous GWAS studies for BCAAs. We additionally detected a rare (MAF = 0.012%, *P* = 2.6 × 10^−13^) missense variant, 19-41414070-A-G (rs771686663), in *BCKDHA* (Fig. 2c) and a rare (MAF = 0.047%, *P* = 6.5 × 10^−29^) splice region variant, 16-31111297-T-A (rs118042732), in *BCKDK* (Fig. 2d). In both cases, the predicted variant effects were directionally consistent with the sign of the GWAS associations (Fig. 2e). The *BCKDHA* missense variant was predicted by AlphaMissense^47^ to be deleterious and was associated with increased BCAA levels. By contrast, the rs118042732 splice region variant in *BCKDK* was predicted by both SpliceAI (score = 0.30) and AlphaGenome (score = 0.34) to lead to splice acceptor loss and was associated with decreased BCAA levels (consistent with BCKDK being a negative regulator of the BCKDH complex) (Fig. 2b). Reassuringly, both *BCKDK* (*P* < 1 × 10^−30^) and *BCKDHA* (*P* < 1 × 10^−13^) were also found to be associated with BCAAs in a parallel effort that performed rare variant burden testing and exome-wide association testing using overlapping UKBB NMR samples^13^, confirming that our rare variant imputation is reliable.

Finally, we detected a novel common variant (7-107837919-T-A, MAF 52%) association at the *DLD* locus (beta = −0.01, *P* = 9.8 × 10^−13^). Thus, we identified GWAS hits for all six key enzymes involved in the catabolism of BCAAs. This illustrates that very large sample sizes are needed to saturate the discovery of key regulators of biological processes owing to either very small effects of some common variants on the target genes (*DLD*) or very low allele frequency of the genetic variants that affect those genes (*BCKDHA* and *BCKDK*). Of note, whereas the GWAS and burden testing analysis by Zoodsma et al.^13^ identified a largely divergent set of genes in this pathway (*BCAT2*, *BCKDK* and *BCKDHA* from burden testing and *BCAT2*, *DLD*, *DBT* and *PPM1K* from GWAS), we detected all six genes from a single analysis. This is consistent with recent reports that the differences between GWAS and burden testing results can be largely explained by differential statistical power^48^.

## Extent of horizontal pleiotropy across metabolic traits

**Fig. 3:**
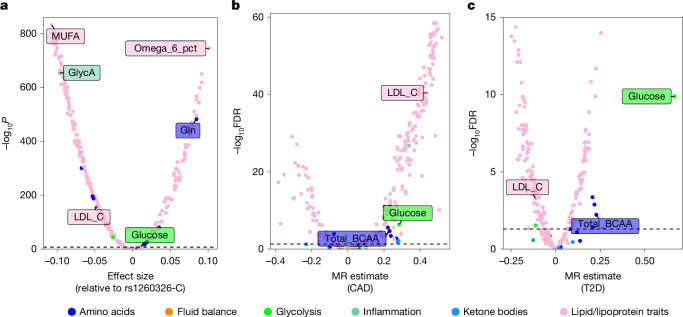
Extent of pleiotropic associations across metabolic traits. **a**, Pleiotropic effects of the *GCKR* missense variant 2-27508073-T-C (rs1260326) on 249 metabolites. The dotted line represents the genome-wide significance threshold (5 × 10^−8^). **b**, Genome-wide Mendelian randomization estimates using all 249 metabolic traits as exposures and CAD as outcome. The *y* axis shows negative log_10_-transformed FDR-adjusted *P* values, with a horizontal line drawn at *P* = 0.05. **c**, Genome-wide Mendelian randomization estimates using all 249 metabolic traits as exposures and T2D as outcome. The *y* axis shows negative log_10_-transformed FDR-adjusted *P* values, with a horizontal line drawn at *P* = 0.05. Source data

**Extended Data Fig. 4:**
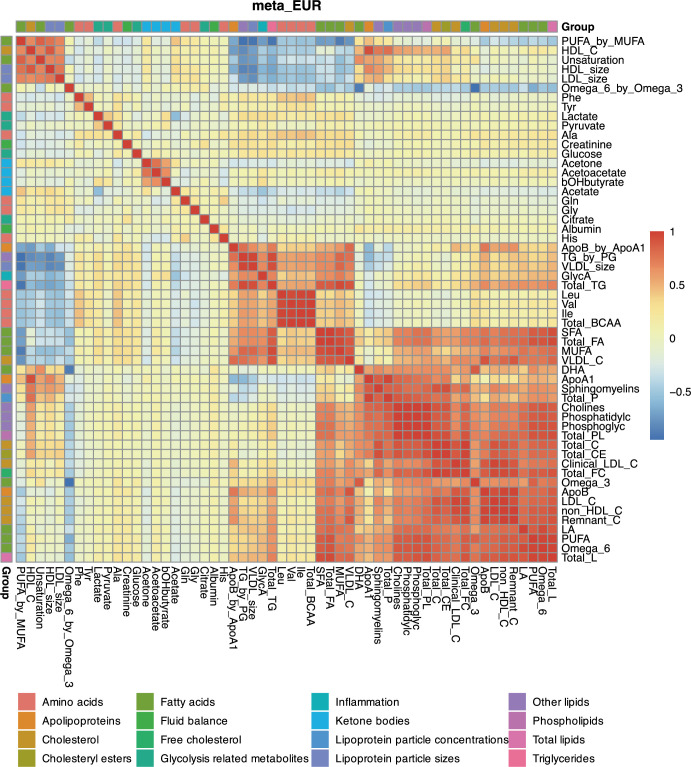
Heatmap of pairwise genetic correlations between metabolic traits in the meta_EUR dataset. The heatmap shows a representative subset of 56 metabolic traits from the main metabolic classes. The complete genetic correlation matrix for all 249 metabolic traits is presented in Supplementary Table 10.

To understand the shared genetic control of various classes of metabolic traits, we explored genetic correlations between all 249 metabolic traits. Although the median genetic correlation across all traits was low (rg = 0.16), there were high genetic correlations between various lipoprotein traits (median rg = 0.52) as well as between other closely regulated metabolites, such as BCAAs (rg = 0.97) (Extended Data Fig. 4 and Supplementary Table 7). To characterize the molecular mechanisms underlying genetic correlations, we focused on the lead variants that were shared (*r*^2^ > 0.8) between metabolic traits. Among the 249 metabolic traits, most lead variants were significantly associated (*P* < 5 × 10^−8^) with multiple metabolites (mean = 10, median = 2). Most prominently, a common missense variant (MAF = 40%) in *GCKR* (2-27508073-T-C, *GCKR*:p.Leu446Pro) was significantly associated (*P* < 5 × 10^−8^) with 229 (out of 249) metabolites (Fig. 3a). However, in many other cases, pleiotropy was restricted to the same class of metabolites, such as the 5-75360714-T-C (rs12916) variant at the *HMGCR* locus, which is associated with multiple lipid traits (Supplementary Fig. 4).

To characterize the effect of pleiotropic genetic effects on interpreting disease associations, we performed genome-wide Mendelian randomization using all 249 metabolic traits as exposures and either CAD^5^ or T2D^1^ as outcomes (see Methods). For CAD, 211 of the 249 tested metabolic traits (85%) yielded significant Mendelian randomization estimates (false discovery rate (FDR) < 5%; Fig. 3b), whereas for T2D (Fig. 3c), the number of significant associations was 157 (63% of tested traits) (Supplementary Table 8). Reassuringly, we recapitulated known causal effects between genetically regulated low-density lipoprotein (LDL) cholesterol and CAD (beta = 0.43, *P* value = 6.02 × 10^−42^) and between glucose and T2D (beta = 0.67, *P* value = 6.3 × 10^−12^). We also detected a known negative association between genetically lower LDL cholesterol and T2D^49^ (beta = −0.11, *P* value = 2.37 × 10^−4^). Finally, we detected genome-wide significant Mendelian randomization estimates between BCAA levels and both CAD and T2D (Fig. 3b,c). However, these genome-wide Mendelian randomization estimates, beyond the known causal effects of LDL and glucose, can be tricky to interpret owing to extensive genetic correlation between the metabolic traits (Extended Data Fig. 4 and Supplementary Table 7), widespread horizontal pleiotropy, and large heterogeneity between the effect estimates from individual variants (Supplementary Table 8).

## Evaluating drug targets

**Fig. 4:**
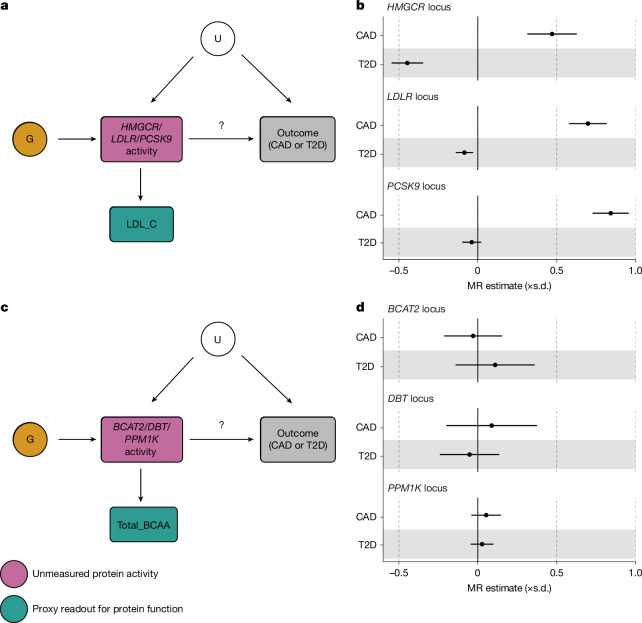
Drug target evaluation with *cis*-Mendelian randomization. **a**, *Cis*-Mendelian randomization using genetic variants G from the *cis* regions of *HMGCR*, *LDLR* and *PCSK9* genes is used to estimate the causal effect of inhibiting the corresponding gene function on risk for T2D^1^ and CAD^5^. LDL cholesterol (LDL_C) is used as a proxy readout for variant effects on *HMGCR*, *LDLR* and *PCSK9* function. **b**, Mendelian randomization estimates from **a**, with 95% confidence intervals. **c**, *Cis*-Mendelian randomization using genetic variants G from the *cis* regions of *BCAT2*, *DBT* and *PPM1K* genes is used to estimate the causal effect of inhibiting the corresponding protein function on risk for T2D^1^ and CAD^5^. Total_BCAA is used as a proxy readout for variant effects on *BCAT2*, *DBT* and *PPM1K* function. **d**, Mendelian randomization estimates from **c**, with 95% confidence intervals. U, unmeasured confounders. Source data

To limit the effect of horizontal pleiotropy, we interrogated a subset of the genome-wide Mendelian randomization associations using a more conservative *cis*-Mendelian randomization approach^50,51^. Instead of capturing average genome-wide effects of circulating metabolic traits, *cis*-Mendelian randomization uses genetic variation in the *cis* region of the target gene to estimate the effect of perturbing gene function on disease risk^51^. If these genes have a direct biological effect on metabolic traits, then we can use the variant effect on those traits as a proxy readout for the (unmeasured) effect of these variants on gene function^11^. First, we focused on three genes with direct effect on regulating plasma LDL cholesterol levels: *LDLR*, *HMGCR* and *PCSK9* (Fig. 4a). In all three cases, we observed robust causal effects of perturbing these genes on CAD risk, as previously reported^9,49^. We then estimated the causal effect of lowering LDL cholesterol through these mechanisms on T2D. At the *HMGCR* locus, we detected a negative association between genetically regulated LDL cholesterol and T2D, which is consistent with previous Mendelian randomization studies as well as large clinical trials demonstrating that statin use is associated with increased T2D risk^49^. Notably, although the effect of genetically regulated LDL cholesterol on CAD risk was even higher at the *LDLR* and *PCSK9* loci, the effect on T2D was strongly attenuated relative to *HMGCR*^49^. This is consistent with clinical trials of *PCSK9* inhibitors that did not detect increased risk of T2D as a side effect^52^. However, our estimates could still be biased by LD between linked causal variants (Supplementary Note 2).

Reassured by the ability of *cis*-Mendelian randomization to rediscover known associations with lipid-lowering drug targets, we next followed up the significant genome-wide Mendelian randomization estimates between BCAAs and both CAD and T2D (Fig. 3b,c). Although the association between genetically regulated BCAAs and T2D has been reported before^6^, genome-wide Mendelian randomization necessarily averages effects across multiple distinct mechanisms, only some of which might influence T2D risk. Furthermore, recent studies have suggested that the genome-wide Mendelian randomization signal between BCAAs and T2D first observed by Lotta et al.^6^ might be confounded by horizontal pleiotropy and reverse causality^13,53,54^. As discussed above, a prominent mechanism that regulates plasma BCAA levels is BCAA catabolism, which is controlled by BCAT2 and the BCKDH complex (Fig. 2a). To clarify the contradictory results obtained from genome-wide Mendelian randomization analysis and motivated by the recent discovery of a clinical candidate BCKDK kinase inhibitor^55^, we sought to assess whether lowering plasma BCAA levels via specific inhibition of the BCKDK kinase could reduce T2D and CAD risk.

The most direct way to assess this would be to perform *cis*-Mendelian randomization with genetic variants in the *cis* region of *BCKDK* as genetic instruments, plasma BCAA levels as a proxy exposure and T2D as outcome. However, the *BCKDK* region lacks strong common variant associations, and the splice donor variant that we detected (Fig. 2d) is too rare to have sufficient power for *cis*-Mendelian randomization. Instead, we focused on *cis* variation near *DBT* and *PPM1K*, two other members of the BCKDH complex (Fig. 2b) that have robust common variant associations. We also included *cis* variation near *BCAT2*, an enzyme that is directly upstream of the BCKDH complex in the BCAA catabolism pathway (Fig. 2a). In all three gene regions, the results were broadly consistent with a null effect of BCKDK inhibition on T2D risk (Fig. 4b). None of these loci had genome-wide significant hits for T2D (Supplementary Figs. 5 and 6). Although some Mendelian randomization method and outcome GWAS combinations (Methods) did yield non-null causal effect estimates, these were not consistent across the three *cis* regions (Supplementary Tables 9–11). Thus, current genetic evidence does not support the idea that *BCKDK* inhibition would have a large beneficial effect on reducing T2D risk.

## Limitations

Our study has several limitations. First, 97% of the samples included in the analysis were of predominantly European genetic ancestries. This skew limited our ability to detect genome-wide significant signals in other genetic ancestry groups and may influence the generalizability of our findings across genetic ancestry groups. As a result, the number of genome-wide significant signals increased by only 1.4% (Table 1) when samples from other UKBB genetic ancestry groups (AFR, AMR, CSA, EAS and MID) were included in the meta-analysis. Secondly, owing to substantial methodological challenges in meta-analysis fine mapping^23^, we performed statistical fine mapping only on the UKBB_EUR subset of the samples. Thus, we probably missed many weaker secondary signals at the genome-wide significant loci. Finally, our analysis was necessarily limited to the 249 (mostly lipoprotein) metabolic traits detectable by the Nightingale Health NMR platform. Previous mass spectrometry-based metabolic trait GWAS studies have profiled a much broader spectrum of metabolites (up to 1,919 traits^56^), but at the cost of significantly lower throughput (at most 19,994 individuals^57^).

Although biobank-scale datasets provide unprecedented power for genetic discovery, they also introduce complexities in interpreting genetic associations due to pervasive pleiotropy. Our results reinforce previous reports of extensive pleiotropy across metabolic trait GWASs^8–10^. Some of this pleiotropy is readily interpretable, such as co-regulation between various lipid traits^9^ or opposing effects between substrates and products of enzymatic reactions^10^. However, given our large sample size, we also detected more cryptic pleiotropic effects, such as the *GCKR* missense variant that was associated with 231 out of 249 tested metabolic traits (Fig. 3a). Such extensive overlaps exemplify that as cohorts grow larger, the detection of pleiotropic signals becomes more pronounced, making it more difficult to disentangle direct and indirect genetic effects. As a result, we caution against interpreting genome-wide Mendelian randomization results as evidence of direct causal effects of tested metabolic traits on the outcomes of interest^58^. *Cis*-Mendelian randomization analyses are potentially less susceptible to these pleiotropic effects, but still require careful consideration of LD and detailed understanding of metabolic pathways to ensure that Mendelian randomization assumptions are met^11,50,51^.

## Conclusion

We have created a comprehensive resource of both common and low-frequency genetic variants associated with 249 metabolic traits in up to 619,372 individuals across multiple ancestry groups. We have demonstrated the utility of the resource for GWAS interpretation via statistical fine mapping, systematic phenome-wide colocalization, low-frequency variant prioritization and *cis*-Mendelian randomization analyses. To ensure that our results can be used as widely as possible, we have publicly released all summary statistics via the GWAS Catalog^59^ and we have also made the association and colocalization results easy to query via two online browsers: https://nmrmeta.gi.ut.ee/ and https://elixir.ut.ee/eqtl/nmr-coloc. Although we were still underpowered to detect many novel associations with rare variants (156 unique lead variants with MAF < 0.1%), our analysis clearly highlights the value of including low-frequency (MAF between 0.1% and 1%) variants in GWAS discovery and interpretation. Whereas 8.6% of the credible sets had low-frequency lead variants, this proportion increased to 19.4% for confidently fine-mapped (PIP > 0.8) variants, suggesting that owing to less extensive LD, low-frequency signals are easier to fine map than common signals. Notably, these low-frequency fine-mapped variants were also twice as likely to be predicted as missense or splice-altering than common fine-mapped variants (23.1% versus 11.5%), thus providing a clear hypothesis about the causal gene and a likely mechanism of action. While traditional multi-cohort GWAS meta-analyses have been limited to common variants^1,5^, low-frequency variants are now routinely included in large biobanks such as FinnGen^28^, MVP^29^ and Pan-UKBB^19^. As a result, 2.5% of our colocalizations involved metabolic trait loci with MAF < 1%. Furthermore, for many common complex diseases, cross-biobank meta-analyses (such as the FinnGen + MVP + UKBB meta-analysis (https://public-mvp-ukbb.finngen.fi/)) are now achieving comparable statistical power to traditional multi-cohort studies, thus making it possible to include low-frequency variants in colocalization and *cis*-Mendelian randomization analyses to reveal disease mechanisms and prioritize drug targets. However, taking full advantage of these low-frequency meta-analysis associations requires accurate fine mapping of conditionally distinct signals, which is currently an active area of research with several novel methods proposed^23,60,61^.

Our results demonstrate that even for well-studied metabolic traits, increasing GWAS sample size can still yield novel discoveries and biological insights. However, as the discovery of associations increases, it is increasingly important to also invest in scalable tools and computational methods to support the interpretation and prioritization of these associations.

## Methods

### Estonian Biobank

The EstBB is a volunteer-based biobank at the Institute of Genomics, University of Tartu^62^. The current EstBB data freeze consists of 212,955 adult (age ≥ 18 years) participants, reflecting the age, sex and geographical distribution of the adult Estonian population, for whom biological samples as well a variety of health-related and demographic information have been collected. All biobank participants have signed a broad informed consent form and their blood sample collection was undertaken across the country between 2002 and 2021^62,63^. The activities of EstBB are regulated by the Human Genes Research Act, which was adopted in 2000 specifically for the operations of EstBB. The Nightingale Health NMR platform was used to generate plasma metabolic trait profiles for all individual samples in the biobank. The assay covers 249 metabolic traits ranging from low molecular weight compounds to lipids and lipoproteins. Individual-level data analysis in EstBB was carried out under ethical approval 1.1-12/624 from the Estonian Committee on Bioethics and Human Research (Estonian Ministry of Social Affairs), using data according to release application 6-7/GI/8988 from the EstBB.

### UK Biobank

The UKBB is a longitudinal biomedical study of approximately half a million participants between 38–71 years of age from the UK^64^. Participant recruitment was conducted on a volunteer basis and took place between 2006 and 2010. Initial data were collected in 22 different assessment centres throughout Scotland, England and Wales. Data collection includes elaborate genotype, environmental and lifestyle data. Blood samples were drawn at baseline for all participants, with an average of 4 h since the last meal (that is, generally non-fasting). NMR metabolic traits (Nightingale Health, quantification library 2020) were measured from EDTA plasma samples (aliquot 3) during 2019–2024 from the entire cohort. Details on the NMR metabolomic measurements in UKBB have been described previously for the first tranche of ~120,000 samples^65^. The UKBB study was approved by the North West Multi-Centre Research Ethics Committee. This research was conducted using the UKBB Resource under application numbers 91233 and 30418.

### NMR data QC and normalization

NMR data generation in the EstBB and UKBB has been previously described^66^. During the quality control of the NMR metabolomics data, we detected a difference between distributions of several metabolic traits (notably Ala and His) driven primarily by spectrometer and batch effect. We removed this unwanted technical variation using the R package ukbnmr in both EstBB and UKBB data^67^. We excluded individuals with more than 5 missing metabolic trait measurements from the cohort, confirmed that none of the 249 metabolic traits had a significant number of missing measurements (8,000 for EstBB, 24,000 for UKBB), and applied inverse normal transformation to each metabolic trait to obtain the final dataset.

### Association testing and meta-analysis

Genotype imputation for the EstBB and UKBB cohorts is described in Supplementary Note 3. We conducted genome-wide association tests for each of the seven genetic ancestry groups separately using regenie v3.1.1^68^, with sex, age, age squared and the top principal components (PCs) of the genotype data used as covariates (PC1–PC10 for EstBB, PC1–PC20 for UKBB). For step 1 (whole-genome model), we used genotype calls for UKBB and genotyping data for EstBB and included variants with a MAF of at least 1%, a minor allele count of at least 20, Hardy-Weinberg equilibrium exact test *P* values of 10^−15^ or less, and maximum per-variant and per-sample missing genotype rates of 0.1. For step 2 (association testing using a linear regression model), we used imputed genotypes and selected variants with a minor allele count of at least 20 and an imputation INFO score of at least 0.6.

We performed two different inverse-variance weighted fixed-effect meta-analyses: meta_EUR on individuals of predominantly European genetic ancestry (EstBB cohort and EUR genetic ancestry group of UKBB), and meta_ALL which encompasses all seven genetic ancestry groups from UKBB and EstBB.

### Genetic correlations

We utilized LD score regression (LDSC)^69,70^ to obtain pairwise genetic correlations for all 249 NMR metabolic traits. Correlations were calculated between biobanks for each metabolic trait and between all metabolic traits in three of the largest datasets (EstBB, UKBB_EUR and meta_EUR) using the European reference panel LD scores from 1000 Genomes, as provided by the authors of LDSC (https://data.broadinstitute.org/alkesgroup/LDSCORE/eur_w_ld_chr.tar.bz2). Genetic correlations were also calculated between traits that were present in both Pan-UKBB lab measurements and NMR measurements in the UKBB_EUR cohort, as well as between two inflammation markers, CRP and GlycA. All Pan-UKBB summary statistics were lifted over to the GRCh38 genome version prior to analysis.

### Lead variant and locus definition

For the common and low-frequency variants (MAF > 0.1%, ~15 million variants), we used the standard genome-wide significance threshold of *P* < 5 × 10^−8^. However, our analysis also included up to 80 million rare variants (MAF < 0.1%) for which the standard *P* value threshold was too lenient. To be conservative, we treated all rare variant tests as independent and used the Bonferroni correction to establish a more stringent significance threshold of *P* < 0.05/80,000,000 (6.25 × 10^−10^). We obtained the set of dataset–metabolic trait–variant triplets by iterating over variants that met these thresholds. The variant with the lowest *P* value was designated as the lead variant within a 2 Mb locus. In each dataset, neighbouring loci were merged into one if their lead variants were in LD with an *r*^2^ of at least 0.05. This was done to prevent very strong association signals (for example, −log_10_*P* > 100) from ‘bleeding’ outside the 2 Mb window. To better evaluate the independence of lead variants, we utilized PLINK v1.90b6.26 to calculate pairwise LD between all lead variants in a single genetic ancestry group, assigning them into shared cross-metabolic trait clusters if *r*^2^ was at least 0.8. The variant with the smallest *P* value was assigned as the lead variant for each cluster.

### Colocalization

We used gpu-coloc to perform large-scale genetic colocalization between metabolic traits and multiple large and publicly accessible repositories and biobanks. The prior probabilities for coloc were set to *p*_1_ = *p*_2_ = 1 × 10^−4^, *p*_12_ = 5 × 10^−6^ as recommended in the gpu-coloc manuscript^26^. For the eQTL Catalogue r7^30,71^, FinnGen r12^28^ and INTERVAL eQTL^31^ datasets, we used fine-mapped SuSiE logarithms of Bayes factors (LBFs) available from these resources directly as input to gpu-coloc. The eQTL Catalogue r7 eQTL and sQTL (quantified by leafCutter) LBFs were downloaded from the eQTL Catalogue FTP Server (https://www.ebi.ac.uk/eqtl/). The FinnGen r12 LBFs were downloaded from the FinnGen website (https://www.finngen.fi/en/access_results). The INTERVAL eQTL LBFs were downloaded from Zenodo (10.5281/zenodo.17956387).

For the other resources, we started with marginal summary statistics and converted these to approximate Bayes factors for colocalization using the algorithm from the approx.bf.estimates function of the coloc^72^ R package (https://github.com/chr1swallace/coloc/blob/main/R/claudia.R#L96). To define loci for colocalization from each GWAS, we started with the variant with the smallest *P* value and defined the region in a ±1 Mb window around that variant as the first locus. We then excluded all variants from the first locus from consideration and proceeded recursively to define additional loci until there were no additional variants with *P* < 5 × 10^−8^ remaining. The Pan-UKBB^19^ association summary statistics for 7,228 traits were downloaded from the https://pan.ukbb.broadinstitute.org/ website. We converted variant coordinates from GRCh37 genome build to GRCh38 with pyliftover^73^ and corrected the reference and alternative alleles with pyfaidx^74^. The marginal summary statistics for the MVP^29^ dataset were downloaded from the public dbGaP FTP server (https://ftp.ncbi.nlm.nih.gov/dbgap/studies/phs002453/phs002453.v1.p1/analyses/GIA/). We corrected the reference and alternative alleles using pyfaidx^74^ and for the qualitative traits, we re-calculated the −log_10_ *P* value from odds ratios and credible intervals, as for some trait–variant pairs the original *P* value provided by the authors was rounded to 0. The UKB-PPP^32^ summary statistics were downloaded from Synapse (https://www.synapse.org/Synapse:syn51364943). For each of the 2,923 proteins, we only used the summary statistics from the *cis* region (±1 Mb) around the corresponding protein coding gene. The summary statistics from the FinnGen + MVP + UKBB meta-analysis were downloaded from https://public-mvp-ukbb.finngen.fi/. The summary statistics from the Suzuki (2024) study^1^ were downloaded from http://www.diagram-consortium.org/downloads.html. The summary statistics from the Aragam (2022) study^5^ were downloaded from the GWAS Catalog (accession GCST90132315).

### Statistical fine mapping

We used SuSiE v0.14.2^75,76^ to identify conditionally distinct association signals around each meta_EUR lead variant that had MAF > 0.1% in the UKBB_EUR subset and excluded variants in the MHC region (chr. 6:28510120–33480577). We utilized the susie_rss method from the susieR R package with prior weights set to null and scaled prior variance set to 0.1. We use LDstore^77^ to calculate in-sample LD matrices for the UKBB_EUR subset of samples on the UKBB’s dnanexus platform. To reduce computational complexity, we divided the genomic regions containing variants of interest into 3 Mb wide windows with a 1 Mb overlap and calculated LD for each. This ensured that each lead variant and the variants up to 500 kb from the lead variant were always contained in at least one LD matrix, except for variants located near chromosome ends or within the excluded MHC region. We imported the LD matrices into R using the rbcor package (https://github.com/mkanai/rbcor). Fine-mapped variants were considered as putative splice-altering variants if at least one of their SpliceAI^24^ or AlphaGenome^25^ donor or acceptor scores across both strands was greater than 0.1. Top 3% of the variants had a SpliceAI score > 0.1 and top 3.8% of the variants had AlphaGenome score > 0.1. To further characterize individual loci, we also performed colocalization between our meta_EUR fine-mapped credible sets and all fine-mapped credible sets available from the Open Targets Platform^35^. We downloaded the credible set files from the Open Targets FTP server (https://ftp.ebi.ac.uk/pub/databases/opentargets/platform/25.09/output/credible_set/).

We used the CLPP method to test if the association signals represented by two credible sets colocalize^78^. We set the colocalization threshold to CLPP > 0.04 as recommended previously^26^.

### Prioritization of functional variants

We first identified all independent lead variants in the meta_EUR analysis that had MAF < 1%. We then narrowed the set down by only including SNPs identified as missense or splice regions variants by the Ensembl Variant Effect Predictor (VEP)^79^ or were predicted to alter splicing by SpliceAI^24^ or AlphaGenome^25^ (at least one of their donor or acceptor scores across both strands was greater than 0.1). This approach identified 91 variants that we were able to assign to putative effector genes (Supplementary Table 6).

### Genome-wide Mendelian randomization

Genome-wide Mendelian randomization studies seek to determine metabolic traits (exposures) that have a causal effect on any number of outcomes (often complex diseases). However, inferences from Mendelian randomization studies are valid only if certain assumptions are met^50,80^. A key assumption of Mendelian randomization is that the genetic variants are associated with the outcome only via the exposure of interest^58^. In practice, this assumption can be challenging to satisfy, because genetic variants can have pleiotropic effects on multiple metabolic traits^8–10^. We performed genome-wide Mendelian randomization between all 249 metabolic traits and two diseases, CAD^5^ and T2D^1^, resulting in a total of 498 analyses. For each metabolic trait, we identified instrumental variables using a greedy LD pruning approach applied to its lead variants with MAF > 1%. This involved: (1) assigning the lead with the lowest *P* value in the initial set to the instrument set; (2) discarding that variant and all variants in LD with it (*r*^2^ < 0.01) from the initial set; and (3) repeating steps A and B until no variants remained in the initial set. For the Mendelian randomization analysis itself, we used multiplicative random-effects inverse-variance weighted Mendelian randomization (IVW-MR) (implemented in MendelianRandomization R package^81^) as recommended in recent guidelines^50^.

### *Cis*-Mendelian randomization

A promising alternative to genome-wide Mendelian randomization is *cis*-Mendelian randomization that focuses the analysis to a specific *cis* region around the target gene of interest^9,51^. While *cis*-Mendelian randomization is less susceptible to horizontal pleiotropy^11,50^, it is limited by the number of independent association signals that can be identified at any gene region, thus requiring very well powered association studies. For the primary *cis*-Mendelian randomization analysis, we included only variants from the ±200 kb region around the gene body of the target gene that had MAF > 1%. For instrument selection, we used the LD information from the UKBB Genomics England imputation (Pan-UKBB EUR subset, *n* = 413,897) and used greedy pruning strategy to only retain variants with *P* < 5 × 10^−8^ and *r*^2^ < 0.01. Although previous studies have used more relaxed *r*^2^ thresholds for LD pruning^9^, we found that our high statistical power required a more stringent filtering to avoid including many variants with low residual LD. We performed the primary *cis*-Mendelian randomization analysis using two pleiotropy-robust methods that we found to perform well in the *cis*-Mendelian randomization context in our previous benchmark study^82^: multiplicative random-effects IVW-MR implemented in the MendelianRandomization R package^81^ and MRLocus^83^. For the IVW-MR method, we also specified ‘weights = delta’. We also repeated the same analysis using MR-Egger^84^.

To further assess the robustness of our Mendelian randomization results, we also tested two other *cis*-Mendelian randomization methods: MR-link-2^85^ and MR-PCA^86^. The advantage of these methods is that instead of requiring instrument selection via LD pruning, they explicitly model the LD between all associated variants in the *cis* region. For MR-link-2 and MR-PCA, all association summary statistics were harmonized to the UK10K genotype ref. ^87^ and both methods were using the default parameters provided by the mr_link_2_standalone.py function: MAF > 0.005, regional definition ±250 kb, and instrument selection threshold *P* < 5 × 10^−8^ and variance explained of the LD matrix of 99%.

### Structural modelling

Models of the three subunits of the BCKDH complex in Fig. 2b were generated using AlphaFold 3 via the AlphaFold Server^88^. Structures of the regulatory proteins BCKDK and PPM1K were retrieved from the AlphaFold Protein Structure Database^89,90^. For clarity in visualization, disordered or unstructured regions at the N and C termini were manually removed. Molecular graphics were performed with UCSF ChimeraX^91^.

### Reporting summary

Further information on research design is available in the Nature Portfolio Reporting Summary linked to this article.

## Online content

Any methods, additional references, Nature Portfolio reporting summaries, source data, extended data, supplementary information, acknowledgements, peer review information; details of author contributions and competing interests; and statements of data and code availability are available at 10.1038/s41586-026-10532-5.

## Supplementary information

Supplementary Information Supplementary Notes 1–3, Supplementary Figs. 1–9 and titles for Supplementary Tables 1–13.

Reporting Summary

Supplementary Tables Supplementary Tables 1–13.

Peer Review File

## Source data

Source Data Figs. 1–4, Extended Data Fig. 3 and Supplementary Figs. 1, 2 and 4

## Figures and Tables

**Extended Data Table 1:**
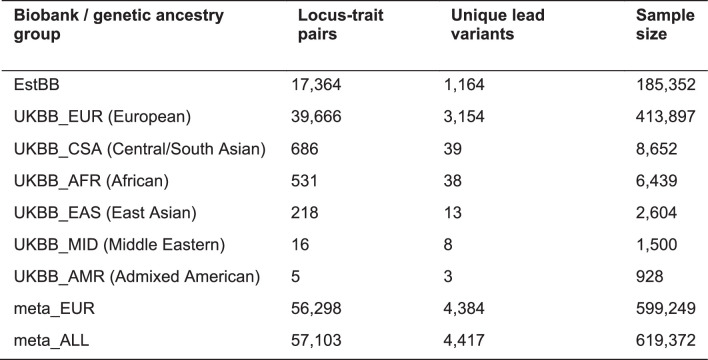
Number of significant locus-metabolic trait pairs and unique lead variants

Compared to Table 1, the significance threshold was set to p < 2 × 10^−10^ (5 × 10^−8^/249) to also account for the 249 tested metabolic traits.

**Extended Data Table 2:**
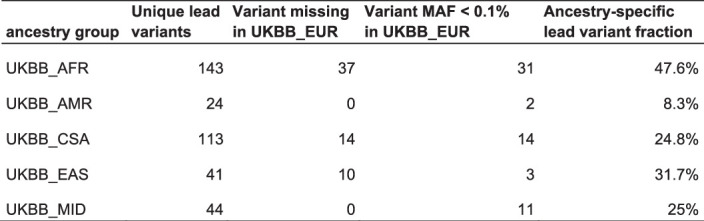
Number of ancestry-specific lead variants detected in each UKBB non-EUR genetic ancestry group

The counts are shown separately for lead variants either completely missing in the UKBB_EUR subset or having MAF < 0.1%.

## Data Availability

Complete genetic ancestry group-specific and meta-analysis association summary statistics from this study can be downloaded from the GWAS Catalog^59^ (accessions GCST90449363–GCST90451603, Supplementary Table 12). GWAS lead variants, fine-mapping credible sets, and colocalization results are available from Zenodo (https://zenodo.org/records/13937265, https://zenodo.org/records/18132538 (ref. ^92^) and https://zenodo.org/records/17945143 (ref. ^93^)). The meta_EUR meta-analysis results can also be viewed in our PheWeb browser at https://nmrmeta.gi.ut.ee/ and the colocalization results can be explored at https://elixir.ut.ee/eqtl/nmr-coloc. The individual-level UKBB data are available for approved researchers through the UKBB data-access protocol (https://www.ukbiobank.ac.uk/enable-your-research/apply-for-access). The individual-level data from Estonia Biobank can be accessed through a research application to the Institute of Genomics of the University of Tartu (https://genomics.ut.ee/en/content/estonian-biobank). Source data are provided with this paper.

## Code availability

Data analysis code is available from https://github.com/ralf-tambets/EstBB-UKBB-metaanalysis/.
