# Supplementary material for: Genetic analysis of circulating metabolic traits in 619,372 individuals
Source: Nature. 2026 May 20;655(8124):971–8. doi: 10.1038/s41586-026-10532-5 (PMC13391353; doi:10.1038/s41586-026-10532-5)
Supplement: Supplementary file 1 — Supplementary Notes 1–3, Supplementary Figs. 1–9 and titles for Supplementary Tables 1–13. [file 41586_2026_10532_MOESM1_ESM.pdf]

nature portfolio

https://doi.org/10.1038/s41586-026-10532-5

Supplementary information

Genetic analysis of circulating metabolic traits in 619,372 individuals

In the format provided by the authors and unedited

Supplementary Materials

Table of Contents

Supplementary Notes **2**
Supplementary Note 1. Example colocalisation at established disease risk loci. 2
Supplementary Note 2. Effect of LD on cis-MR estimates. 3
Supplementary Note 3. Genotype imputation. 3

Description of Supplementary Tables **5**

Supplementary Figures **6**
Supplementary Fig. 1 | Relationship between heritability and the number of genome-wide significant hits. 6
Supplementary Fig. 2 | Comparison of shared lead variant betas in our European-ancestry meta-analysis (meta_EUR) and results presented by Karjalainen *et al*. 7
Supplementary Fig. 3 | Detected metabolite trait associations with low-frequency variants. 8
Supplementary Fig. 4 | Pleiotropic effects of the *HMGCR* locus lead variant rs12916 on many lipid-related metabolites. 9
Supplementary Fig. 5 | Association signals for BCAAs, lipoprotein traits and T2D near *BCAT2*. 10
Supplementary Fig. 6 | Regional association plots for T2D and valine in the *cis* regions of (A) *DBT* and (B) *PPM1K*. 11
Supplementary Fig. 7 | Colocalisation between HMGCR sQTL, LDL cholesterol and CAD. 12
Supplementary Fig. 8 | HMGCR sQTL signal in the Alasoo_2018 dataset. 13
Supplementary Fig. 9 | Examples of colocalising eQTL - metabolic trait - disease triplets. 14

Supplementary References **15**

Supplementary Notes

Supplementary Note 1. Example colocalisation at established disease risk loci.

Colocalisation with CAD and T2D. To assess the quality and scope of our resource, we focused on two common complex diseases with an established metabolic component: CAD and T2D. CAD was involved in 37 colocalisation clusters that contained a median of 177 metabolic traits (range 1-227). For example, a splicing QTL (rs12916, 5-75360714-T-C) affecting the inclusion of exon 13 of 3-hydroxy-3-methylglutaryl-CoA reductase (*HMGCR*) colocalised with both CAD and LDL cholesterol as well as 127 other metabolic traits from our analysis (cluster 351, **Supplementary Figs. 7-8, Supplementary Table 5**). Similarly, a previously established and fine mapped liver-specific eQTL for *SORT1* (1-109274968-G-T, PIP = 0.99)[1] colocalised with 182 metabolic traits (including LDL cholesterol) and CAD risk, as well as with several additional cardiovascular outcomes across the three independent biobank studies (cluster 61, **Supplementary Fig. 9**). In contrast, T2D was involved in 127 clusters that contained a median of 16 metabolic traits (range 1-228). As an example, a known pancreatic islet-specific enhancer variant 3-123346931-A-G[2] was detected as an eQTL in the PISA pancreatic islet dataset[3] for *ADCY5* and colocalised with both glucose, T2D and other diabetes-related traits (cluster 207, **Supplementary Fig. 9**). Together, these examples highlight the utility of our meta-analyses results and demonstrate that large-scale colocalisation integrating metabolic and molecular traits enables the identification of tissue-specific regulatory mechanisms at known disease associated loci.

GlycA as a biomarker of chronic inflammation. Next, we focused on the glycoprotein acetyls (GlycA) - a complex metabolic trait that has been linked to chronic inflammation[4,5]. There were 227 colocalisation clusters that involved GlycA as one of the metabolic traits. In FinnGen+UKBB+MVP meta-analysis, we detected at least one colocalisation with GlycA for 105 (out of 330) disease endpoints. The most common disease endpoints colocalising with GlycA were dyslipidemias (30 clusters), T2D (16 clusters), coronary atherosclerosis (15 clusters), hypertension (13 clusters) and gout (13 clusters), followed by many other diseases with fewer colocalisations. For example, a known variant in the *IL6R* gene[6] (1-154454494-A-C, rs2228145) was associated with GlycA in meta_EUR ($p = 4.6 \times 10^{-26}$) and colocalised with coronary atherosclerosis, ischemic heart disease, and several other cardiovascular endpoints (cluster 74). Furthermore, an established risk locus for inflammatory bowel disease (IBD)[7] (21-39094542-G-A, rs9808651) colocalised with GlycA and an eQTL for *ETS2* transcription factor in stimulated monocytes (cluster 141). We next investigated if GlycA GWAS could provide added value over C-reactive protein (CRP) - another inflammatory biomarker for which large-scale GWAS studies exist[8,9]. We found that in 43/227 clusters, GlycA signal colocalised with CRP as profiled by Pan-UKBB. Similarly, the genetic correlation between GlycA and CRP in the UK Biobank was 0.53, which was much lower than the average of 0.96 for matched lab measurements from Pan-UKBB (**Supplementary Table 13**, see **Methods**). These results indicate that GlycA and CRP capture complementary aspects of chronic inflammation, and

suggest that our GlycA GWAS results can help to interpret a wide range of traits and diseases involving chronic inflammation.

Supplementary Note 2. Effect of LD on cis-MR estimates.

One limitation of *cis*-MR is that due to the focus on a narrow *cis*-region for the selection of genetic instruments, it can be sensitive to LD between independent causal variants[10,11]. For example, although the association signals between LDL cholesterol and T2D at the *HMGCR* locus do not colocalise with each other[12], the two lead variants are in moderate LD ($r^2 = 0.53$), which could likely bias our *cis*-MR estimates for T2D (**Fig. 4a**) at this locus. The impact of LD is likely to become more pronounced as the sample size and power of GWAS studies increase. For example, even though the lead variant at the *BCAT2* locus (19-48800958-C-T) has a genome-wide significant ($p < 5\times10^{-8}$) association with 30 lipid traits, this seems to be entirely driven by low LD ($r^2 = 0.08$) with a neighbouring *FUT2* locus lead variant (19-48703417-G-A) (**Supplementary Fig. 5**).

Supplementary Note 3. Genotype imputation.

Estonian Biobank. All EstBB participants have been genotyped at the Core Genotyping Lab of the Institute of Genomics, University of Tartu, using Illumina Global Screening Array v1.0, v2.0 and v3.0. Samples were genotyped and PLINK format files were created using Illumina GenomeStudio v2.0.4. Individuals were excluded from the analysis if their call-rate was < 95%, if they were outliers of the absolute value of heterozygosity (> 3 standard deviations from the mean) or if sex defined based on heterozygosity of the X chromosome did not match sex in phenotype data[13]. Before imputation, variants were filtered by call-rate < 95%, HWE p-value < 1×10^{-4} (autosomal variants only), and minor allele frequency < 1%. Genotyped variant positions were lifted over from GRCh37 to GRCh38 with Picard. Phasing was performed using the Beagle v5.4[14]. Imputation was performed with Beagle v5.4 (beagle.22Jul22.46e.jar) using default settings. Dataset was split into batches of 5,000 variants. A population specific reference panel consisting of 2,695 whole genome sequencing samples[13] was utilised for imputation and standard Beagle hg38 recombination maps were used. Based on principal component analysis, samples that did not belong to the predominantly European genetic ancestry group were removed. Duplicate and monozygous twin detection was performed with KING 2.2.7[15], and one sample was removed out of the pair of duplicates.

UK Biobank autosomes. Genotype imputation for the UK Biobank (UKBB) autosomal data was conducted using a high-coverage whole genome sequencing reference panel (342 million autosomal variants) from 78,195 individuals from the Genomics England (GEL) project. Reference panel construction and UK Biobank imputations have been described previously (UKBB data field 21008)[16].

Briefly, the UKBB SNP array data consisted of 784,256 autosomal variants. Initially, 113,515 sites identified by previous centralised UK Biobank analysis as failing quality control were removed, along with an additional 39,165 sites failing a Hardy–Weinberg equilibrium test on

409,703 GBR samples, with a p-value threshold of 10^{-10}. The resulting SNP array data were mapped from the GRCh37 to GRCh38 genome build using the GATK Picard LiftOver tool. Alleles with mismatching strands but matching alleles were flipped. A further 495 sites were removed due to incompatibility between the two reference genomes, resulting in a final SNP array incorporating 631,081 autosomal variants used for phasing and imputation.

Haplotype estimation of the SNP array data, a prerequisite for imputation, was carried out one chromosome at a time using SHAPEIT4 v4.2.2[17] without a reference panel, utilising the full set of UK Biobank samples. SHAPEIT4 was run with its default 15 Markov chain Monte Carlo iterations and 30 threads. Autosomal imputation using the GEL reference panel was conducted with IMPUTE5[18] (v.1.1.4). The SNP array data were divided into 408 consecutive and overlapping chunks of approximately 5 megabases (Mb) each, with a 2.5 Mb buffer across the genome using the Chunker program in IMPUTE5. Each chunk was further divided into 24 sample batches, each containing 20,349 samples. IMPUTE5 was run on each of the 9,792 subsets using a single thread and default settings. The resulting imputed genotype dosages are stored in BGEN format, and phasing information is stored in VCF format.

UK Biobank X chromosome. As the UKBB genotypes imputed by Genomics England did not include the X chromosome, we used the TOPMed r2 imputation for the X chromosome (UKBB data field 21007). Imputation was performed using the TopMed Imputation Server[19]. The data were divided into 10 Mb chunks, and each chunk underwent several checks to ensure validity. These checks included verifying the inclusion of variants in the reference panel, ensuring a sufficient overlap with the reference panel, and maintaining an adequate sample call rate. Chunks that did not meet these criteria were excluded from further analysis. Overall, quality control methods employed by the TopMed Imputation Server were slightly more conservative than those employed by GEL and thus the sample size for each sub-population decreased by roughly 0.5% (final sample sizes: AFR - 6,411; AMR - 925; CSA - 8,627; EAS - 2,595; EUR - 412,523; MID - 1,491). Genotype phasing was performed with Eagle2[20] and imputation was conducted with mimimac4[19]. After imputation, all chunks of each chromosome were merged into a single file. For chromosome X, additional checks were performed to verify ploidy and ensure the accuracy of mixed genotypes. The chromosome was split into three regions (PAR1, non-PAR, PAR2) for phasing and imputation, and these regions were later merged into a complete chromosome X file.

Description of Supplementary Tables

Supplementary Table 1: List of all 249 metabolic traits included in the analysis.

Supplementary Table 2: Genetic correlation for all metabolic traits between the Estonian Biobank and UK Biobank.

Supplementary Table 3: Estimated heritability of each metabolic trait from LD score regression.

Supplementary Table 4: Fine mapped variants with PIP > 0.8.

Supplementary Table 5: All pairwise colocalisation from selected colocalisation clusters discussed in the main text.

Supplementary Table 6: Low-frequency and rare missense and splice-altering lead variants.

Supplementary Table 7: Pairwise genetic correlation between all metabolic traits in the meta_EUR meta-analysis.

Supplementary Table 8. Results from genome-wide MR analysis for T2D and CAD.

Supplementary Table 9. Results from *cis*-MR analysis with random-effect IVW-MR and MR-Egger.

Supplementary Table 10: Results from *cis*-MR analysis with MRLocus.

Supplementary Table 11: Results from *cis*-MR analysis with MR-link-2.

Supplementary Table 12: Download paths for GWAS summary statistics hosted at the GWAS Catalog.

Supplementary Table 13. Genetic correlation between laboratory measurements in Pan-UKBB and NMR measurements in UKBB_EUR.

Supplementary Figures

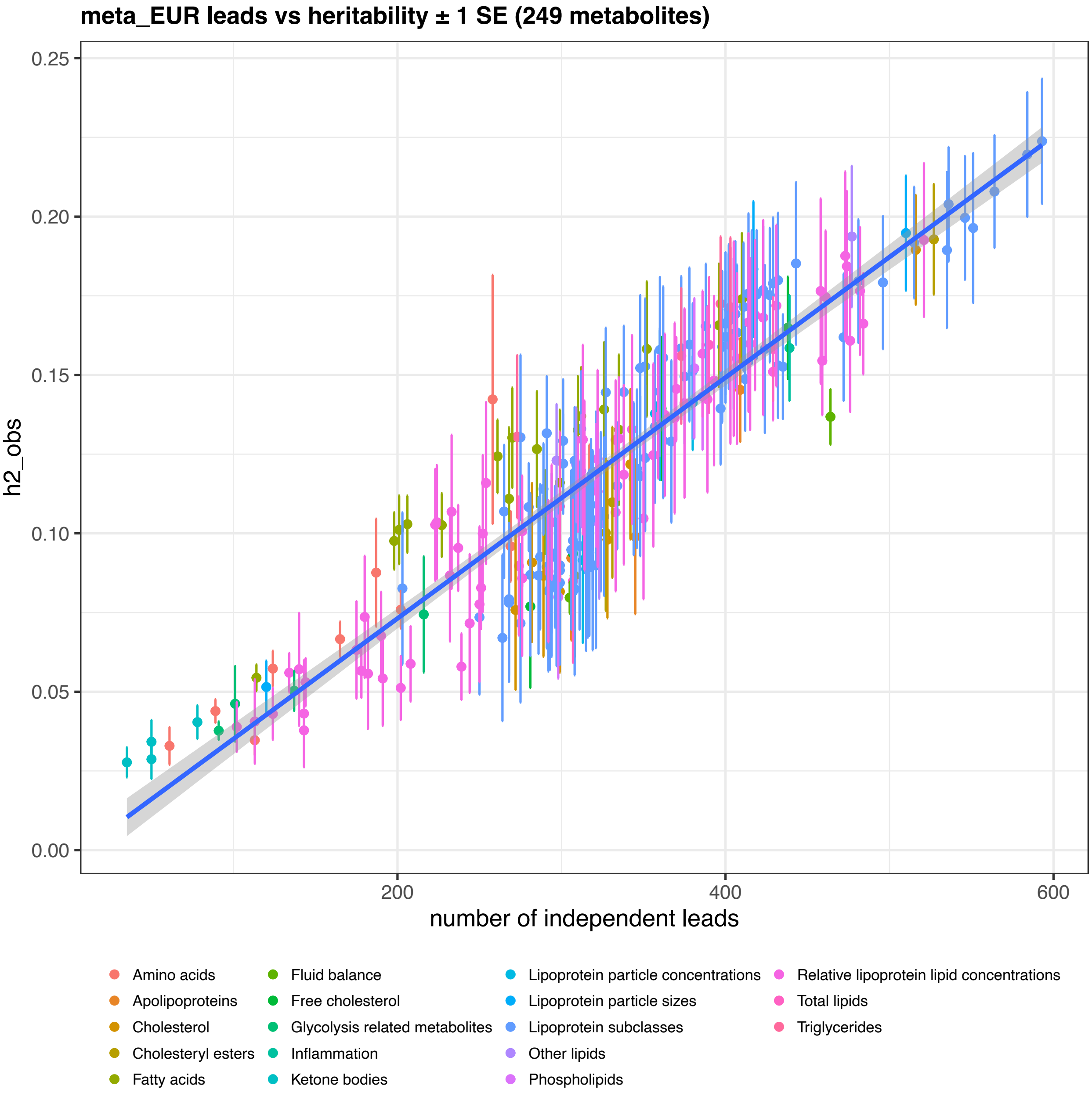

Supplementary Fig. 1 | Relationship between heritability and the number of genome-wide significant hits.

Heritability was estimated with LD score regression for each 249 metabolic traits in the meta_EUR meta-analysis. The error bars represent 95% confidence intervals. h2_obs – heritability.

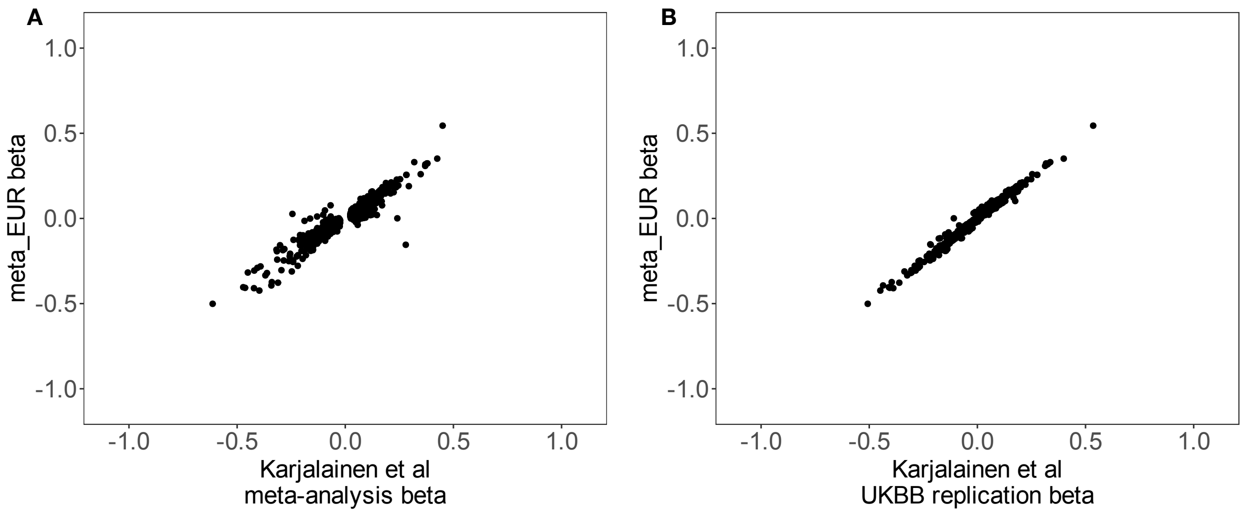

Supplementary Fig. 2 | Comparison of shared lead variant betas in our European-ancestry meta-analysis (meta_EUR) and results presented by Karjalainen *et al*.

(**A**) Scatter plot of GWAS lead variant effect sizes from Karjalainen *et al* main analysis (n = 137k, 33 cohorts) and our meta_EUR. (**B**) GWAS lead variant effect sizes from Karjalainen *et al* UK biobank replication (n = 100k) and our meta_EUR meta-analysis. Even though Karjalainen *et al* included 3,701 samples from the Estonian Biobank, these were older samples profiled in 2011- 2012 that were excluded from our meta-analysis due to significant batch effects. Thus, there is no sample overlap between our meta-analysis and the primary analysis conducted by Karjalainen *et al* (panel **A**). The ~100,000 UK Biobank samples used for replication by Karjalainen *et al* were also part of our meta-analysis, explaining the extremely high concordance in GWAS effect sizes.

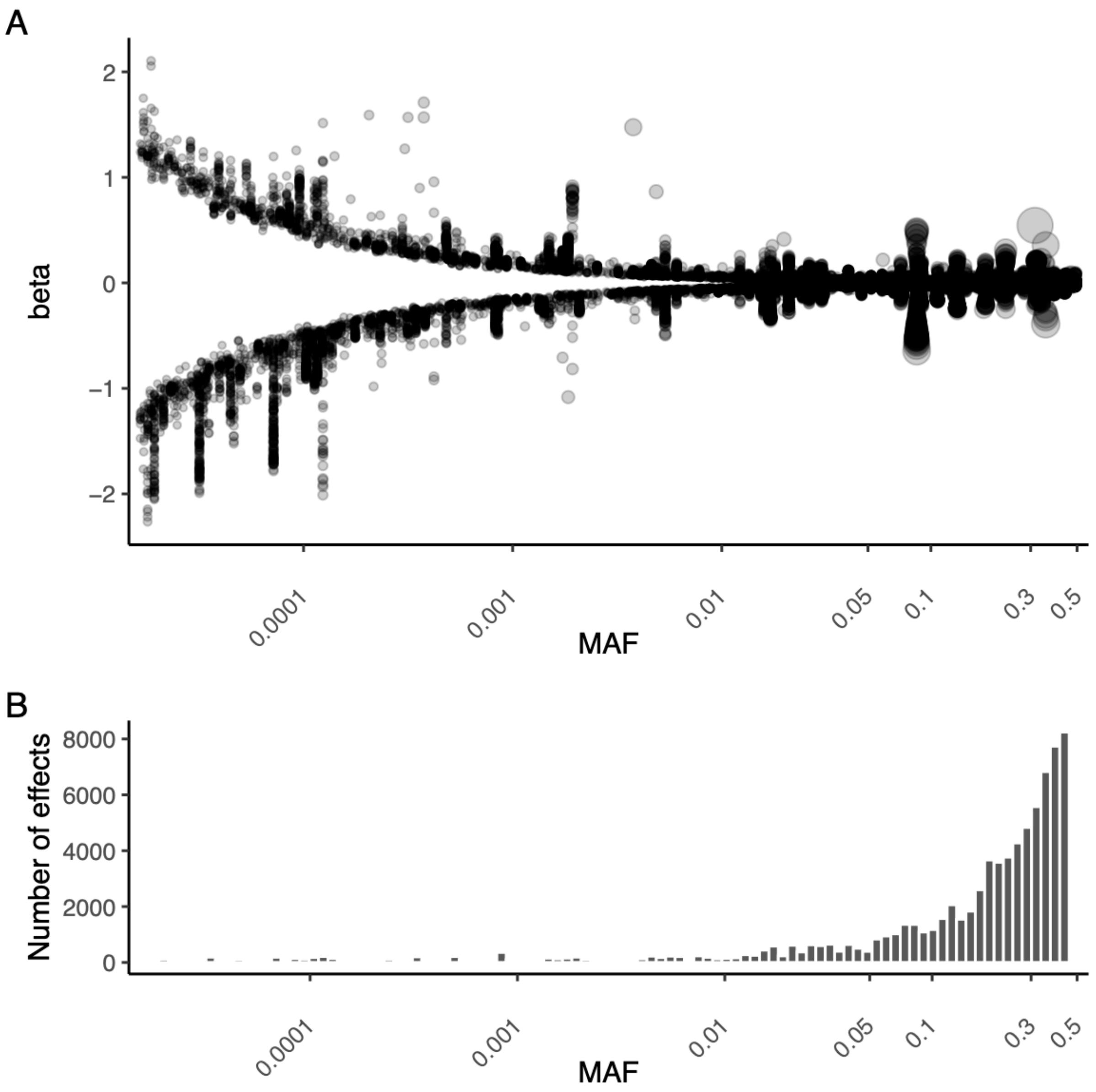

Supplementary Fig. 3 | Detected metabolite trait associations with low-frequency variants.

(**A**) Relationship between the lead variant minor allele frequency (MAF) and effect size (beta). Each dot signifies the lead variant (+/- 1Mb window) from each locus-trait pair (meta_EUR). The size of each dot has been scaled by $-\log_{10}$ p-value. (**B**) Number of detected significant associations in relation to the lead variant MAF in meta_EUR analysis.

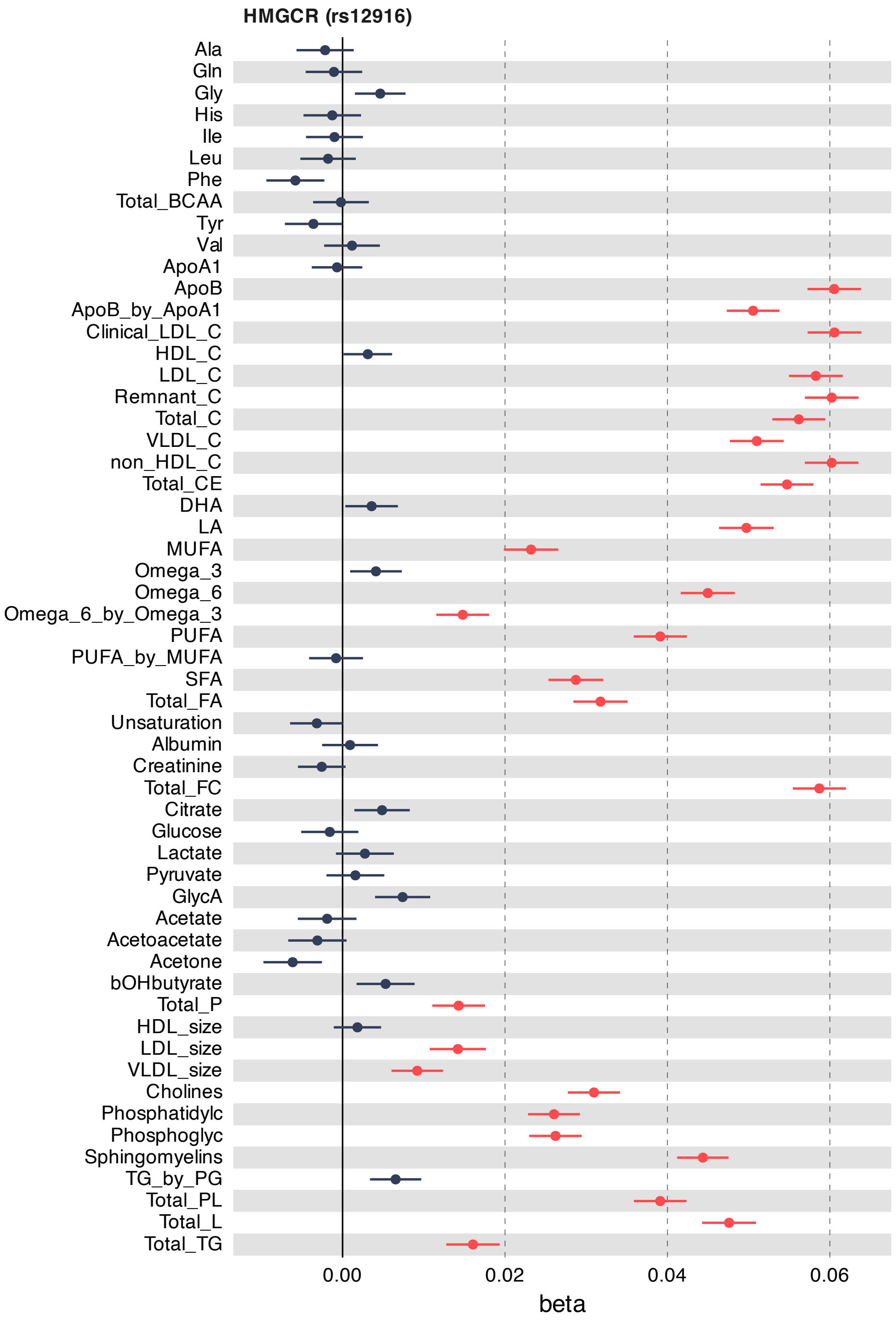

Supplementary Fig. 4 | Pleiotropic effects of the *HMGCR* locus lead variant rs12916 on many lipid-related metabolites.

The forest plot shows a representative subset of 56 metabolic traits from the main metabolic classes. The points show the standardised GWAS effect size (beta) and error bars show the 95% confidence intervals.

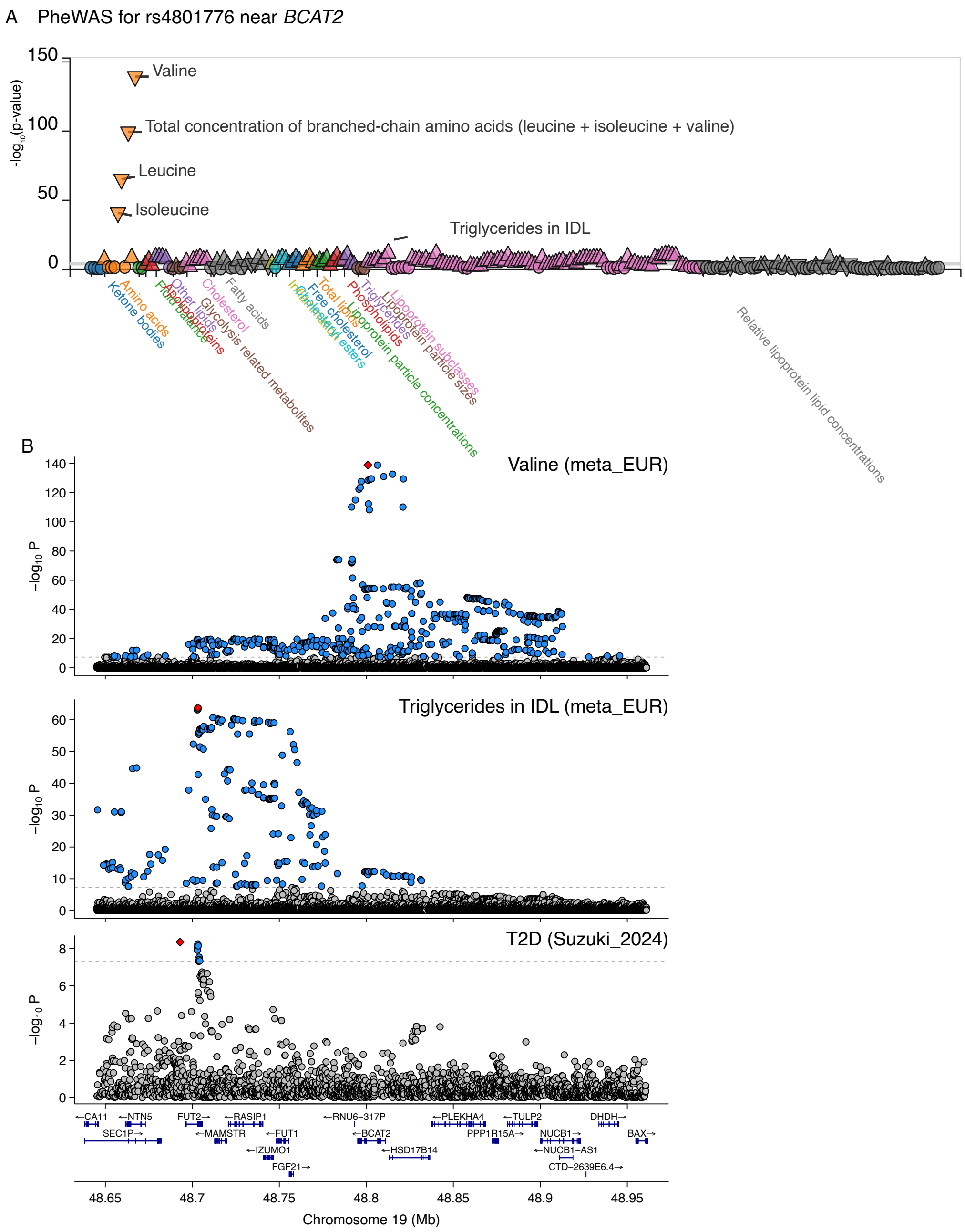

Supplementary Fig. 5 | Association signals for BCAAs, lipoprotein traits and T2D near *BCAT2*.

(**A**) PheWAS plot for the valine lead variant (rs4801776) in the intron of *BCAT2*. In addition to very strong associations with all three branched-chain amino acids, we also see genome-wide significant associations with various lipid traits. (**B**) Regional association plots for Valine, Triglycerides in IDL and T2D in the *BCAT2* region. The association between rs4801776 and lipid traits seems to be driven by an independent lipid signal near the *FUT2* gene that has low LD (r^2 = 0.08) with the valine lead variant.

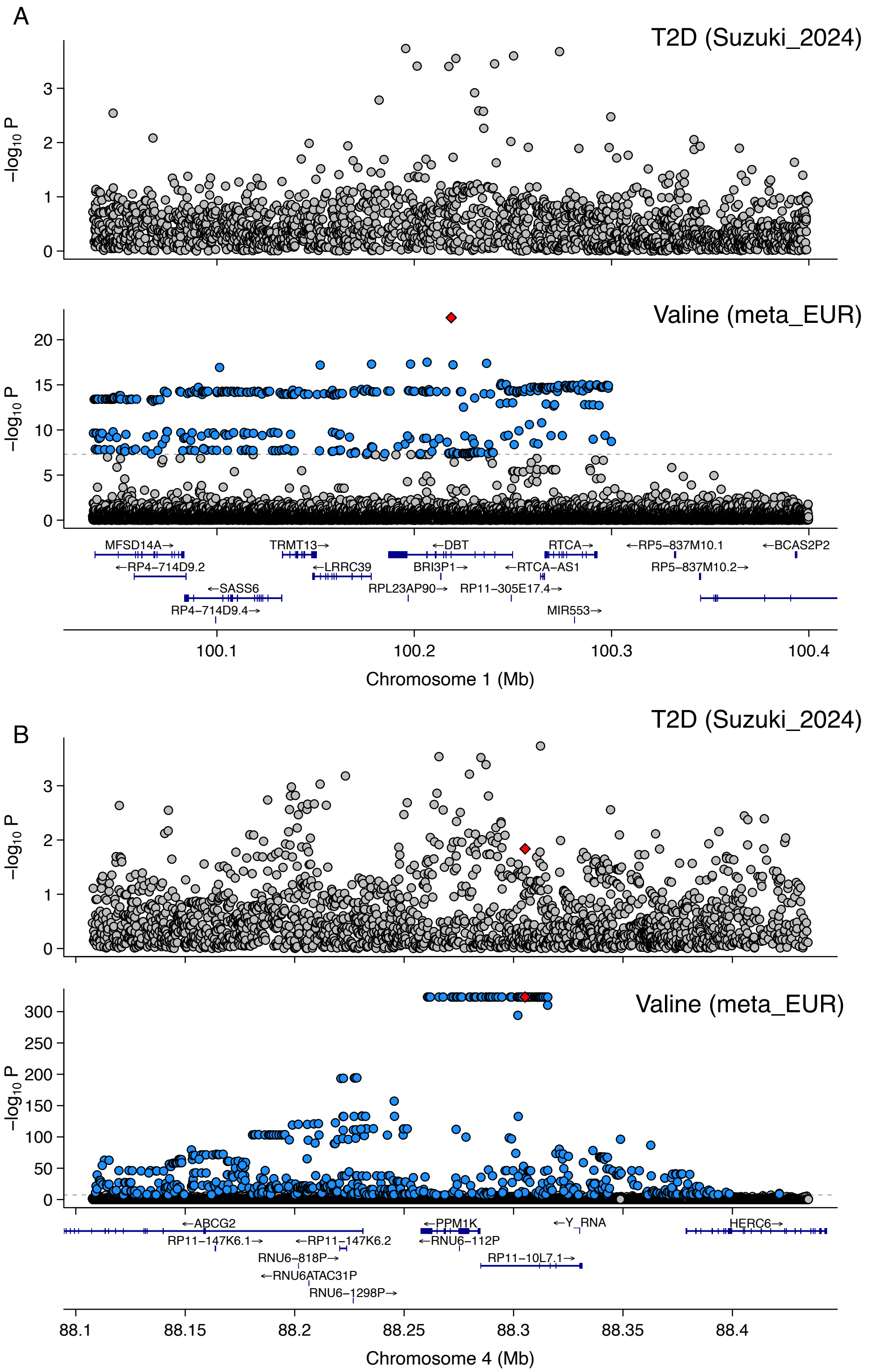

Supplementary Fig. 6 | Regional association plots for T2D and valine in the *cis* regions of (A) *DBT* and (B) *PPM1K*.

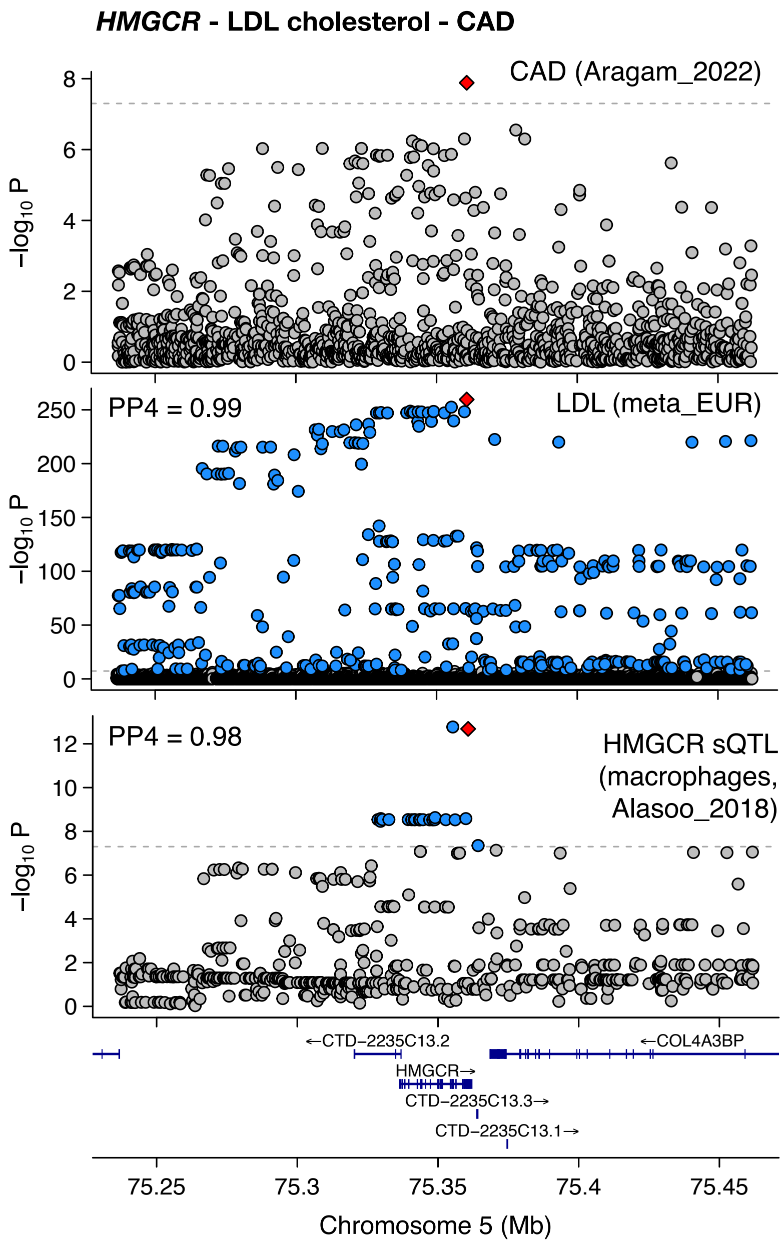

Supplementary Fig. 7 | Colocalisation between HMGCR sQTL, LDL cholesterol and CAD.

HMGCR is a known target for statin therapy to lower circulating LDL levels and reduce CAD risk[21]. The three panels show regional association plots for CAD (Aragam *et al*, 2022[22]), LDL cholesterol and *HMGCR* exon 13 skipping sQTL from Alasoo_2018[23]. PP4 values show pairwise colocalisation posterior probabilities between CAD and the other two traits. In the sQTL analysis, statistical fine mapping prioritised 5-75355259-A-G as the most likely causal variant (posterior inclusion probability (PIP) = 0.64), a finding that has also been validated experimentally[24]. Consistent with a recent report, we did not detect colocalisation between the *HMGCR* sQTL signal and T2D, suggesting that T2D association at this locus involves additional genetic mechanisms[12].

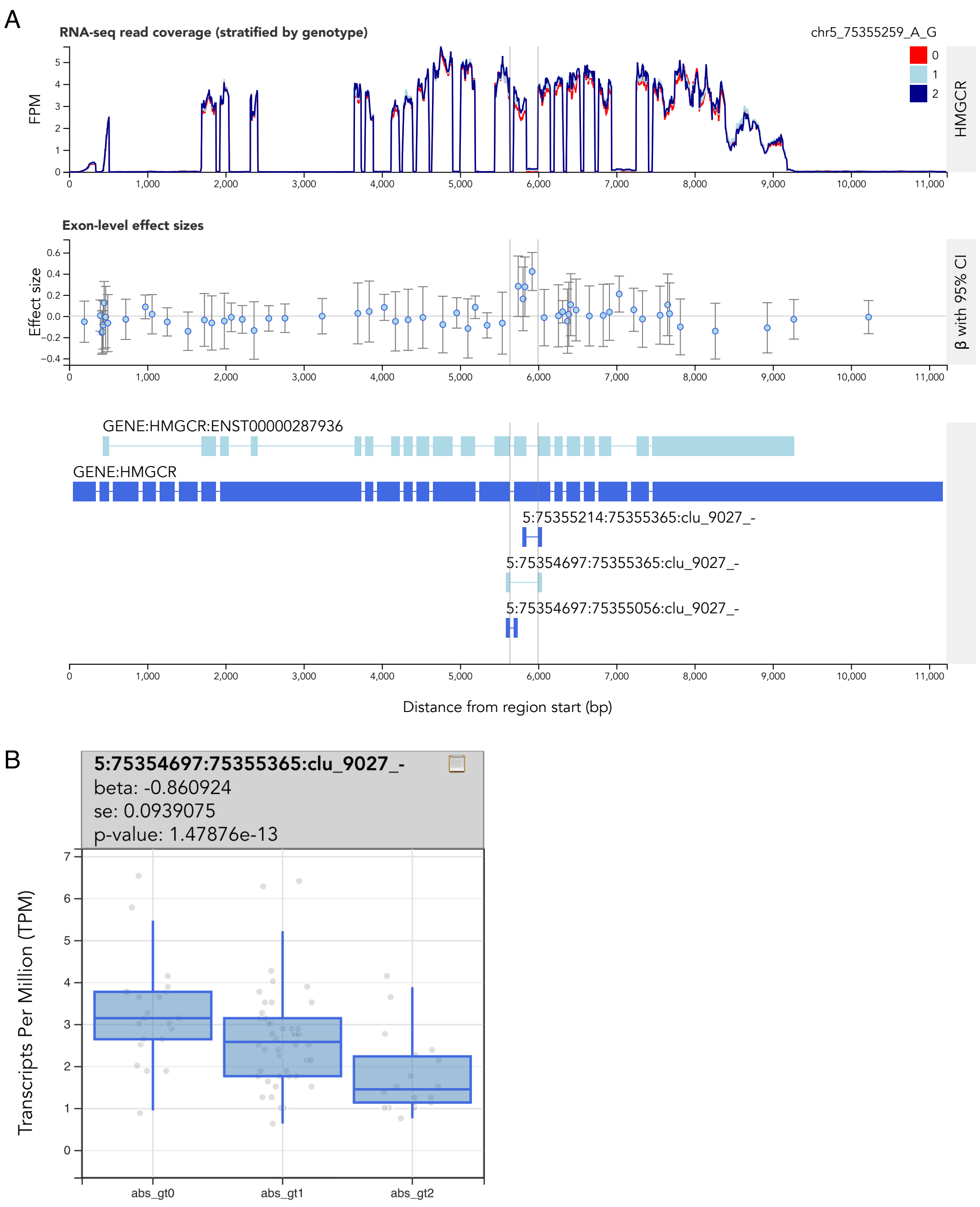

Supplementary Fig. 8 | HMGCR sQTL signal in the Alasoo_2018 dataset.

(**A**) RNA-seq read coverage across the HMGCR gene stratified by the genotype of the lead sQTL variant (5-75355259-A-G). (**B**) Usage of the exon 13-skipping splice junction stratified by the genotype of the lead sQTL variant. Interactive visualisation is available here.

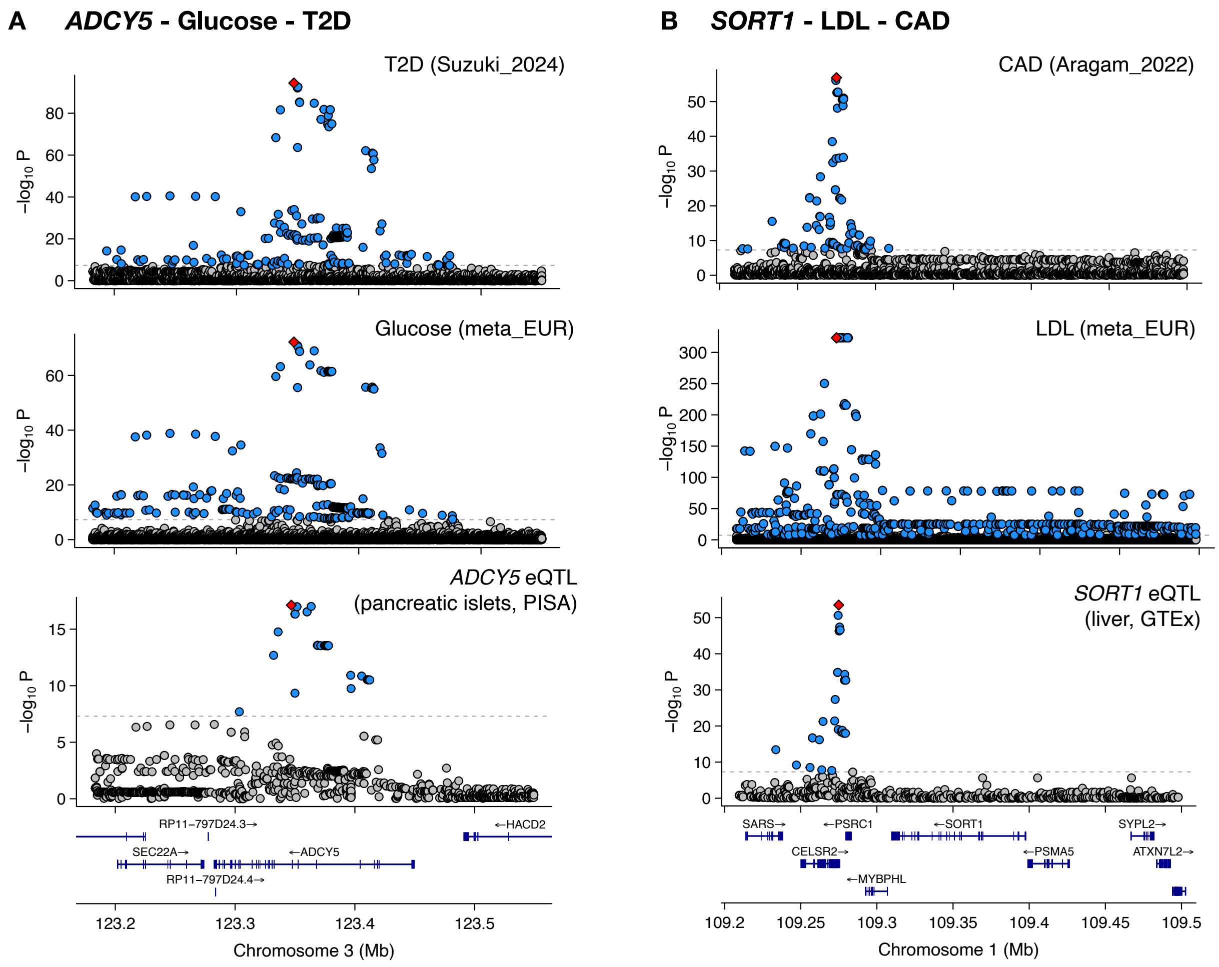

Supplementary Fig. 9 | Examples of colocalising eQTL - metabolic trait - disease triplets.

(**A**) Colocalisation between *ADCY5* eQTL in pancreatic islets, plasma glucose and T2D GWAS.
(**B**) Colocalisation between *SORT1* eQTL in the liver, plasma LDL cholesterol and CAD.

Supplementary References

1. Musunuru, K. *et al.* From noncoding variant to phenotype via SORT1 at the 1p13 cholesterol locus. *Nature* **466**, 714–719 (2010).
2. Roman, T. S. *et al.* A type 2 diabetes-associated functional regulatory variant in a pancreatic islet enhancer at the ADCY5 locus. *Diabetes* **66**, 2521–2530 (2017).
3. Alonso, L. *et al.* TIGER: The gene expression regulatory variation landscape of human pancreatic islets. *Cell Rep.* **37**, (2021).
4. Ritchie, S. C. *et al.* The Biomarker GlycA Is Associated with Chronic Inflammation and Predicts Long-Term Risk of Severe Infection. *Cell Syst* **1**, 293–301 (2015).
5. Otvos, J. D. *et al.* GlycA: A composite nuclear magnetic resonance biomarker of systemic inflammation. *Clin. Chem.* **61**, 714–723 (2015).
6. IL6R Genetics Consortium Emerging Risk Factors Collaboration *et al.* Interleukin-6 receptor pathways in coronary heart disease: a collaborative meta-analysis of 82 studies. *Lancet* **379**, 1205–1213 (2012).
7. Stankey, C. T. *et al.* A disease-associated gene desert directs macrophage inflammation through ETS2. *Nature* 1–10 (2024).
8. Said, S. *et al.* Genetic analysis of over half a million people characterises C-reactive protein loci. *Nat. Commun.* **13**, 2198 (2022).
9. Karczewski, K. J. *et al.* Pan-UK Biobank genome-wide association analyses enhance discovery and resolution of ancestry-enriched effects. *Nat. Genet.* **57**, 2408–2417 (2025).
10. Zuber, V. *et al.* Combining evidence from Mendelian randomization and colocalization: Review and comparison of approaches. *Am. J. Hum. Genet.* **109**, 767–782 (2022).
11. Wu, Y. *et al.* Integrative analysis of omics summary data reveals putative mechanisms underlying complex traits. *Nat. Commun.* **9**, 918 (2018).
12. Hwang, S. *et al.* Human genetics suggests differing causal pathways from HMGCR inhibition to coronary artery disease and type 2 diabetes. *Int. J. Epidemiol.* **55**, dyaf223 (2026).
13. Mitt, M. *et al.* Improved imputation accuracy of rare and low-frequency variants using population-specific high-coverage WGS-based imputation reference panel. *Eur. J. Hum. Genet.* **25**, 869–876 (2017).
14. Browning, B. L., Tian, X., Zhou, Y. & Browning, S. R. Fast two-stage phasing of large-scale sequence data. *Am. J. Hum. Genet.* **108**, 1880–1890 (2021).
15. Manichaikul, A. *et al.* Robust relationship inference in genome-wide association studies. *Bioinformatics* **26**, 2867–2873 (2010).
16. Shi, S. *et al.* A Genomics England haplotype reference panel and imputation of UK Biobank. *Nat. Genet.* **56**, 1800–1803 (2024).
17. Delaneau, O., Zagury, J.-F. & Marchini, J. Improved whole-chromosome phasing for disease and population genetic studies. *Nat. Methods* **10**, 5–6 (2013).
18. Rubinacci, S., Delaneau, O. & Marchini, J. Genotype imputation using the Positional Burrows Wheeler Transform. *PLoS Genet.* **16**, e1009049 (2020).
19. Das, S. *et al.* Next-generation genotype imputation service and methods. *Nat. Genet.* **48**, 1284–1287 (2016).
20. Loh, P.-R. *et al.* Reference-based phasing using the Haplotype Reference Consortium panel. *Nat. Genet.* **48**, 1443–1448 (2016).

21. Ference, B. A. *et al.* Variation in PCSK9 and HMGCR and Risk of Cardiovascular Disease and Diabetes. *N. Engl. J. Med.* **375**, 2144–2153 (2016).
22. Aragam, K. G. *et al.* Discovery and systematic characterization of risk variants and genes for coronary artery disease in over a million participants. *Nat. Genet.* **54**, 1803–1815 (2022).
23. Alasoo, K. *et al.* Shared genetic effects on chromatin and gene expression indicate a role for enhancer priming in immune response. *Nat. Genet.* **50**, 424–431 (2018).
24. Burkhardt, R. *et al.* Common SNPs in HMGCR in micronesians and whites associated with LDL-cholesterol levels affect alternative splicing of exon 13. *Arterioscler. Thromb. Vasc. Biol.* **28**, 2078–2084 (2008).
