# Supplementary material for: Genetic analysis of circulating metabolic traits in 619,372 individuals
Source: Nature. 2026 May 20;655(8124):971–8. doi: 10.1038/s41586-026-10532-5 (PMC13391353; doi:10.1038/s41586-026-10532-5)
Supplement: Supplementary file 2 — Reporting Summary [file 41586_2026_10532_MOESM2_ESM.pdf]

nature portfolio

Corresponding author(s): Kaur Alasoo, Priit Palta

Last updated by author(s): Apr 1, 2026

Reporting Summary

Nature Portfolio wishes to improve the reproducibility of the work that we publish. This form provides structure for consistency and transparency in reporting. For further information on Nature Portfolio policies, see our Editorial Policies and the Editorial Policy Checklist.

Statistics

For all statistical analyses, confirm that the following items are present in the figure legend, table legend, main text, or Methods section.

n/a	Confirmed	
☐	☒	The exact sample size (n) for each experimental group/condition, given as a discrete number and unit of measurement
☐	☒	A statement on whether measurements were taken from distinct samples or whether the same sample was measured repeatedly
☐	☒	The statistical test(s) used AND whether they are one- or two-sided<br>*Only common tests should be described solely by name; describe more complex techniques in the Methods section.*
☐	☒	A description of all covariates tested
☐	☒	A description of any assumptions or corrections, such as tests of normality and adjustment for multiple comparisons
☐	☒	A full description of the statistical parameters including central tendency (e.g. means) or other basic estimates (e.g. regression coefficient) AND variation (e.g. standard deviation) or associated estimates of uncertainty (e.g. confidence intervals)
☐	☒	For null hypothesis testing, the test statistic (e.g. F, t, r) with confidence intervals, effect sizes, degrees of freedom and P value noted<br>*Give P values as exact values whenever suitable.*
☐	☒	For Bayesian analysis, information on the choice of priors and Markov chain Monte Carlo settings
☒	☐	For hierarchical and complex designs, identification of the appropriate level for tests and full reporting of outcomes
☐	☒	Estimates of effect sizes (e.g. Cohen's d, Pearson's r), indicating how they were calculated

Our web collection on statistics for biologists contains articles on many of the points above.

Software and code

Policy information about availability of computer code

Data collection	No software was used for data collection.
Data analysis	Genome-wide association tests were conducted using regenie v3.1.1. Custom inverse-variance weighted fixed-effect metaanalysis code was used, available at https://github.com/ralf-tambets/EstBB-UKBB-metaanalysis/. LD score regression (LDSC) v1.0.1 was employed to obtain pairwise genetic correlations for all 249 NMR metabolites. PLINK v1.90b6.26 was utilized to calculate pairwise LD between lead variants. susieR v0.14.276, LDStore v2.0 and rbcor (available at https://github.com/mkanai/rbcor) were used to perform statistical fine mapping. Colocalisation analyses were conducted using gpu-coloc, available at https://github.com/mjesse-github/gpu-coloc/. Missense and splice region variants were identified using Ensembl Variant Effect Predictor, SpliceAI and AlphaGenome APIs. "MendelianRandomization" R package v0.10.0 was used for genome-wide MR and cis-MR. In addition, MRLocus (v0.0.25), MR-link-2 (v1.0.0) and MR-PCA (v1.0.0) were used to assess the robustness of cis-MR results. Models of the three subunits of the BCKD complex in Figure 3b were generated using AlphaFold 3 via the AlphaFold Server. Molecular graphics were performed with UCSF ChimeraX v1.11.

For manuscripts utilizing custom algorithms or software that are central to the research but not yet described in published literature, software must be made available to editors and reviewers. We strongly encourage code deposition in a community repository (e.g. GitHub). See the Nature Portfolio guidelines for submitting code & software for further information.

Data

Policy information about availability of data

All manuscripts must include a data availability statement. This statement should provide the following information, where applicable:

- Accession codes, unique identifiers, or web links for publicly available datasets
- A description of any restrictions on data availability
- For clinical datasets or third party data, please ensure that the statement adheres to our policy

Complete genetic ancestry group-specific and meta-analysis association summary statistics from this study can be downloaded from the GWAS Catalog (accessions GCST90449363 - GCST90451603, Supplementary Table 12). GWAS lead variants, fine mapping credible sets, and colocalisation results are available from Zenodo (URLs https://zenodo.org/records/13937265, https://zenodo.org/records/18132538, and https://zenodo.org/records/17945143). The meta_EUR meta-analysis results can also be viewed in our PheWeb browser at https://nmrmeta.gi.ut.ee/ and the colocalisation results can be explored at https://elixir.ut.ee/eqtl/nmr-coloc. The individual-level UK Biobank data are available for approved researchers through the UK Biobank data-access protocol (https://www.ukbiobank.ac.uk/enable-your-research/apply-for-access). The individual-level data from Estonia Biobank can be accessed through a research application to the Institute of Genomics of the University of Tartu (https://genomics.ut.ee/en/content/estonian-biobank).

Research involving human participants, their data, or biological material

Policy information about studies with human participants or human data. See also policy information about sex, gender (identity/presentation), and sexual orientation and race, ethnicity and racism.

Reporting on sex and gender	Sex was used as a covariate in genome-wide association tests and was assigned based on chromosome information in the genotyping data. Gender data was not taken into account. We did not perform sex-stratified GWAS as other GWAS studies of the same metabolic traits have have identified highly concordant genetic effects between sexes (e.g. Zoodsma et al, 2025).
Reporting on race, ethnicity, or other socially relevant groupings	We grouped UK Biobank participants into six genetic ancestry groups using information provided by the Pan-UKBB project. Both Estonian Biobank and UK Biobank consist predominantly of individuals of European descent.
Population characteristics	The Estonian Biobank is a volunteer-based biobank of approximately 200, 000 adult (18+) participants, reflecting the age, sex and geographical distribution of the adult Estonian population.<br>The UK Biobank is a longitudinal biomedical study of approximately half a million participants between 38-71 years old from the United Kingdom.
Recruitment	Recruitment of the Estonian Biobank participants was conducted on a volunteer basis. All biobank participants have signed a broad informed consent form and their blood sample collection was undertaken across the country between 2002 and 2021.<br><br>UK Biobank participant recruitment was conducted on a volunteer basis and took place between 2006 and 2010 in 22 different assessment centers throughout Scotland, England, and Wales.
Ethics oversight	Analysis of Individual level from the Estonian Biobank was carried out under ethical approval 1.1-12/624 from the Estonian Committee on Bioethics and Human Research (Estonian Ministry of Social Affairs), using data according to release application 6-7/GI/8988 from the Estonian Biobank.<br>The UK Biobank study was approved by the North West Multi-Centre Research Ethics Committee. This research was conducted using the UK Biobank Resource under application numbers 91233 and 30418.

Note that full information on the approval of the study protocol must also be provided in the manuscript.

Field-specific reporting

Please select the one below that is the best fit for your research. If you are not sure, read the appropriate sections before making your selection.

☒ Life sciences ☐ Behavioural & social sciences ☐ Ecological, evolutionary & environmental sciences

For a reference copy of the document with all sections, see nature.com/documents/nr-reporting-summary-flat.pdf

Life sciences study design

All studies must disclose on these points even when the disclosure is negative.

Sample size	Sample size refers to the number of participants in each genetic ancestry group. For autosomes, the sample sizes were 928 for UKBB_AMR; 1,500 for UKBB_MID; 2,604 for UKBB_EAS; 6,439 for UKBB_AFR; 8,652 for UKBB_CSA; 185,352 for EstBB; and 413,897 for UKBB_EUR. For X chromosome, the sample sizes were 925 for UKBB_AMR; 1,491 for UKBB_MID; 2,595 for UKBB_EAS; 6,411 for UKBB_AFR; 8,627 for UKBB_CSA; 185,352 for EstBB; and 412,523 for UKBB_EUR.<br><br>Previous GWAS studies of metabolic traits have demonstrated that these sampe sizes are sufficient to identify robust associations.
Data exclusions	Estonian Biobank individuals were excluded from the analysis if their genotyping call-rate was < 95%, if they were outliers of the absolute value of heterozygosity (> 3SD from the mean) or if sex defined based on heterozygosity of X chromosome did not match sex in phenotype

	data. To reduce the impact of population stratification on GWAS association analysis, principal component analysis of the genotype data was used to exclude a small number of participants that did not cluster with the other predominantly European ancestry individuals. Duplicate and monozygous twin detection was performed and one sample was removed out of the pair of duplicates.<br>The UK Biobank analysis was restricted to 434,020 individuals that were confidently assigned to one of six genetic ancestry groups by the Pan-UKBB project and had high quality NMR metabolic trait data available.<br>In both UK Biobank and Estonian Biobank, we excluded individuals with more than 5 missing metabolite measurements from the cohort.
Replication	We did not perform explicit replication of novel associations detected in the meta-analysis but we observed highly concordant genetic associations in the Estonian Biobank and UK Biobank (median genetic correlation 0.91). Also, 95% of the loci from a previous GWAS meta-analysis conducted on non-overlapping samples (Karjalainen et al, 2024) replicated in our study.
Randomization	Randomization was not relevant as no new primary data was collected in this study. We analyzed existing metabolic trait and genotype data data from the Estonian Biobank and the UK Biobank.
Blinding	The analysis conducted in this study did not involve group allocations were blinding would have been relevant and/or possible.

Reporting for specific materials, systems and methods

We require information from authors about some types of materials, experimental systems and methods used in many studies. Here, indicate whether each material, system or method listed is relevant to your study. If you are not sure if a list item applies to your research, read the appropriate section before selecting a response.

Materials & experimental systems

n/a	Involved in the study
☒	☐ Antibodies
☒	☐ Eukaryotic cell lines
☒	☐ Palaeontology and archaeology
☒	☐ Animals and other organisms
☒	☐ Clinical data
☒	☐ Dual use research of concern
☒	☐ Plants

Methods

n/a	Involved in the study
☒	☐ ChIP-seq
☒	☐ Flow cytometry
☒	☐ MRI-based neuroimaging

Plants

Seed stocks	*Report on the source of all seed stocks or other plant material used. If applicable, state the seed stock centre and catalogue number. If plant specimens were collected from the field, describe the collection location, date and sampling procedures.*
Novel plant genotypes	*Describe the methods by which all novel plant genotypes were produced. This includes those generated by transgenic approaches, gene editing, chemical/radiation-based mutagenesis and hybridization. For transgenic lines, describe the transformation method, the number of independent lines analyzed and the generation upon which experiments were performed. For gene-edited lines, describe the editor used, the endogenous sequence targeted for editing, the targeting guide RNA sequence (if applicable) and how the editor was applied.*
Authentication	*Describe any authentication procedures for each seed stock used or novel genotype generated. Describe any experiments used to assess the effect of a mutation and, where applicable, how potential secondary effects (e.g. second site T-DNA insertions, mosiacism, off-target gene editing) were examined.*
