# Supplementary material for: Genetic analysis of circulating metabolic traits in 619,372 individuals
Source: Nature. 2026 May 20;655(8124):971–8. doi: 10.1038/s41586-026-10532-5 (PMC13391353; doi:10.1038/s41586-026-10532-5)
Supplement: Supplementary file 4 — Peer Review File [file 41586_2026_10532_MOESM4_ESM.pdf]

nature portfolio

Peer Review File

Genome-wide association study for circulating metabolic traits in 619,372 individuals

Corresponding Author: Dr Kaur Alasoo

Any redactions in this file are there to maintain patient confidentiality, the confidentiality of unpublished data, or to remove third-party material.

This file contains all reviewer reports in order by version, followed by all author rebuttals in order by version.

Version 0:

Reviewer comments:

Referee #1

(Remarks to the Author)
In this manuscript, Drs. Kaur Alasoo, Priit Palta, Ralf Tambets and colleagues present the world's largest genetic mapping of metabolites to date - outlining a massive-scale GWAS meta-analysis incorporating 249 circulating metabolites across up to 619,372 people in the Estonian Biobank and the UK Biobank. The authors integrate both common and less commonly-occurring (MAF<1%) variants into their analyses, identifying 88,604 genetic associations with metabolites - many of them novel (e.g., 27% novel associations for 3-Hydroxybutyrate and 85% novel associations for Lactate). The authors then used these metabolite QTLs (mQTLs) for downstream genetic colocalization analyses, focusing on coronary artery disease and type 2 diabetes - highlighting how inhibition of BCAA levels by modulating the BCAA catabolism pathway is not likely to reduce risk of type 2 diabetes. The authors make their results fully accessible via the GWAS Catalog and a dedicated interactive portal, providing the scientific community with a valuable resource for hypothesis generation and therapeutic target prioritization. Overall, the paper is very well written and the study's sample size is exceptional - affording the authors enough power to increase the discovery rate of its most recent comparator (Karjalainen et al., Nature, 2024) by approximately ten-fold. The authors' observations that leveraging high-quality imputations of rare variants can increase detection rates above and beyond those reported using rare variant burden testing via exome- or whole-genome analyses is also quite remarkable and will be of interest to the broader genetics community. Despite the manuscript's strengths, I have several suggestions for improvements before it should be considered for publication in Nature.

My major pieces of feedback for the manuscript are as follows:

• In an attempt to differentiate this study from prior published metabolite QTL GWAS papers, the authors tested for associations with relatively rare genetic variants (i.e., those with minor allele counts <1%). However, they benchmarked their findings against a recent pre-print from Claudia Langenberg and team, which performed rare variant burden mapping of metabolic traits leveraging sequencing data readily available in UK Biobank. As such, the study's novelty mainly lies in the *scale* at which it is investigating rare variant associations - not necessarily the fact that it is investigating rare variant associations with metabolites in general. This should be clarified earlier in the manuscript. Furthermore - and perhaps more importantly - the value of this rare variant mapping for therapeutic target discovery or understanding of disease mechanisms is not entirely clear in this iteration of the manuscript. The authors themselves acknowledge that rare mQTLs are difficult to implement as instruments for causal mapping (line 357: "…the BCKDK region lacks strong common variant associations and the low-frequency splice donor variant that we detected is too rare to have sufficient power for cis-MR"). Thus, what is the ultimate translational impact of rare variant mapping of metabolic traits? It appears - at least in this version of the manuscript - that its value is limited to greater understanding of basic biological mechanisms, but not necessarily a greater understanding of disease mechanisms or therapeutic targets.

• Although the authors provide relatively interesting examples of how mQTLs can help us understand potential therapeutic mechanisms for Type 2 Diabetes (T2D) and coronary artery disease (CAD), I believe that they have missed an opportunity to present a more comprehensive mapping of causal associations between metabolites and complex illnesses by focusing on only two exemplary indications wherein metabolites likely play the broadest, most complex roles in disease pathophysiology. The authors themselves acknowledge that their GPU-accelerated re-implementation of the coloc algorithm

is approximately 100-fold faster than the original R implementation; as such, I see no technical hurdles to conducting a more comprehensive mapping, which may reveal dozens of additional novel insights into disease mechanisms. The cornerstone exemplar in the current version of the paper - an assessment of the potential therapeutic effect of BCKDK inhibition on reducing T2D risk - is noteworthy but insufficient to fully illustrate the power of the mQTL dataset generated by the authors. Integrating genetic associations with a broader collection of complex, commonly occurring illnesses will strengthen the paper and increase its relevance for the broad readership of Nature.

• The authors state that, "Our publicly available results will provide a valuable resource for GWAS interpretation and target prioritisation studies." I agree that this will be a valuable resource, and I commend the authors for already making the underlying summary statistics available via GWAS Catalog and a dedicated, interactive browser. This is a major strength of the paper. However, to fully optimize the utility of these data, the browser would benefit from three moderate updates:
○ First, when users search by gene (e.g., APOE), the query produces a table listing "top p-value in gene" and "phenotype". It omits the lead variant tagging each association, which is an important component to maintain for ease of interpretation.
○ Second, each phenotype contains an embedded hyperlink which seems as if it is meant to take the user to a new page after they click on it - either to the primary source of association (e.g., the paper in question) or to a new table showing the top variants and genes associated with that phenotype. Instead, it produces a LocusZoom Manhattan plot mapping hits at the general genetic locus in GWAS Catalog. This is not particularly intuitive, as after clicking on the phenotype, one would expect to either produce a full Manhattan plot showing all associations for that phenotype from chromosome 1 through 23, or alternatively to be taken to a new page listing all established genetic associations with that trait. Instead, clicking on the phenotype produces a LocusZoom which remains fixed at the original queried gene (e.g., APOE), which is confusing and counter-intuitive.
○ Third, as an optional but highly valuable update, I would recommend integrating causal mapping results into the browser. This is especially relevant if the authors decide to expand beyond their initially limited focus on T2D and CAD.

• Approximately 3.3% (20,123) of the samples assayed in this study are from individuals of non-European ancestries. The authors should be commended for conducting ancestry-specific analyses in five non-European sub-cohorts of these 20,123 individuals - including people of African, Admixed American, Middle Eastern, East Asian and Central/South Asian ancestries. However, the main text provides only a superficial overview of insights gleaned from these analyses, mainly providing a numeric breakdown of the total number of unique lead variants identified in each non-EUR cohort (with as few as 24 lead variants identified in Admixed Americans and as many as 143 lead variants identified in Africans). Were any mQTLs enriched in non-Europeans and/or depleted in Europeans? Was the median MAF enrichment of lead primary ancestry associations enriched for certain non-European ancestries (e.g., AFR) compared to others (e.g., CSA) when using the EUR cohorts as a benchmark? Are there any unique or interesting insights to be gleaned from these non-European participants? This section could benefit from further details.

Other, minor comments on the manuscript are as follows:

• The authors colocalized 87,373 signals from their meta-analysis of metabolic traits in Europeans with splicing QTLs and expression QTLs extracted from the eQTL catalog. Did the authors also consider integrating protein QTLs into this pipeline? If not, a short summary / justification would be helpful - perhaps this relates to the currently incomplete coverage of proteomic technologies in blood plasma (capturing only <50% of the circulating proteome). However, considering the controversies surrounding the use of eQTLs for fine-mapping of genetic associations with disease (discussed in greater detail by Mostafavi et al., Nature Genetics, 2023 https://www.nature.com/articles/s41588-023-01529-1), it is worth considering integrating of multiple QTL types beyond eQTLs.

• The study leverages data generated using NMR metabolomics, primarily provided by Nightingale Health. While the NMR method offers high reproducibility, a standardized pipeline and the ability to scale to large biobank studies such as UKB and Estonia Biobank, it has limitations compared with other metabolic profiling techniques, such as mass spectrometry-based methods offered by companies like Metabolon. The latter technique can capture upwards of 4-5 times as many metabolites, with greater coverage of the major metabolic pathways as well as rarer metabolites, isomers and exogenous compounds. The limitations of NMR should be noted in the Discussion section.

• The Discussion section ends somewhat abruptly by concluding an overview of the study's limitations. I suspect that this is due to limitations on word count; however, a sentence or two to conclude the manuscript would be appropriate to include at the end of the Discussion.

• The manuscript is extremely well written overall but as is expected with any first draft, I noticed some minor grammatical or stylistic issues - for example:
○ Abstract: Consider changing "can be greatly improved by detailed understanding" to "can be improved by a gaining a greater understanding" or something similar.
○ Lines 36-27: "…can be greatly improved by detailed understanding of molecular consequences of genetic variation" - please include a, "the" before "molecular consequences".
○ Line 403: "…genetic variants from PPM1K, DBT and BCAT2 gene regions" - please add a "the" before "PPM1K".

Referee #2

(Remarks to the Author)
This study conducted a large-scale genome-wide association study (GWAS) meta-analysis of 249 circulating metabolic

traits measured by nuclear magnetic resonance (NMR) spectroscopy, involving up to 619,372 individuals from the UK Biobank and Estonian Biobank. The analysis identified 88,604 associations between genetic loci and metabolic traits across 8,774 independent genomic regions. The authors performed GPU-facilitated systematic genetic colocalization analyses to explore shared genetic signals between metabolic traits, gene expression and splicing quantitative trait loci (QTLs), and the health outcomes of coronary artery disease (CAD) and type 2 diabetes (T2D). Additionally, cis-Mendelian randomization (MR) analyses were conducted to investigate potential causal relationships between metabolic traits and these complex diseases. The study highlighted extensive pleiotropy among metabolic traits, reinforcing previous findings, and provided insights into the biological pathways underlying these associations.

The main strength of this study compared to previous GWAS of metabolic traits is its significantly larger sample size (approximately 3-5 times larger than earlier studies and 40% larger than a parallel recent study described in an online preprint), enabling greater statistical power to detect novel genetic associations, including low-frequency variants. The inclusion of genetic associations with imputed X-chromosome variants also is a strength. Additionally, the integration of genetic colocalization and Mendelian randomization analyses facilitates deeper biological insights into the causal relationships between metabolic traits and complex diseases. The manuscript is also generally very well written and organized and the authors have produced a potentially impressive resource for the research community.

The study's weaknesses include limited genetic diversity, as most participants were of European ancestry, potentially limiting discovery and generalizability, but, more notably, limited novelty of biological insights described. The main problem in this respect is not the analytic approach, but rather the metabolomic platform which is biased and limited as well as dominated by blood lipid related traits and other metabolites that make up well established and well understood biological pathways studied for 50 years or more already (e.g. lipids, lipoprotein subclasses, fatty acid composition, inflammation markers, and various low-molecular metabolites like amino acids, ketones, and glycolysis-related metabolites). In this context, the newly identified genetic links within these well-established pathways is largely predictable as are the genetic correlation analyses showing pervasive pleiotropy.

Specific comments by section as well as suggestions for improvement are included below:

1) Abstract
a) Soften language: you predict that lowering BCAAs won't help T2D, didn't "find" it

2) Intro
a) 94: be consistent in "metabolite" vs. "metabolic trait". The latter is probably preferable, and it seems to be the term used more often in the manuscript.

3) Results
a) 125: What does "overlapping set of UKBB samples" mean? What the studies being overlapped with the UKB?
b) 135: Great that you included the X chromosome in the analysis. That's so often excluded.
c) 156: Might be good to clarify what you mean specifically by "colocalized". Is it PP4 > 0.8? Do you include high PP3 probabilities too? Do you use a different threshold? I'm sure it's in the methods section, but it'd be helpful to mention briefly in passing here so readers know how to interpret these statements about colocalizations.
d) 185/186: how do you define "strongest metabolic association"? Based on P value from the GWAS? Coloc PP4 value for colocalization with the eQTL signal? Something else?
e) 190: I'm not sure I follow the logic here. The GWAS for CAD and GlycA were performed in different data sets. Plus, CAD is a binary outcome and GlycA is continuous. I'm not sure how the effect sizes here are directly comparable, nor how the effect estimate from the GlycA GWAS being smaller than the effect from the CAD GWAS implies the causal direction. Can you elaborate more on the logic here?
f) 237+: This is a arguable the most novel finding here, highlighting the associations you found in other parts of the BCAA metabolic pathway by looking into rare variants.
g) 281: Just my personal preference, but I think Figure S6 would make sense as a main figure.

4) Discussion
a) 409: This whole paragraph is focused on the Zoodsma preprint, pointing out ways the current study is superior to Zoodsma et al. This paragraph seems like maybe it would be appropriate in a message to an editor to justify this paper over the other, but in this manuscript, it just feels out of place and oddly critical of another lab's work. I'd prefer to see this paragraph removed.
b) 436: They should at least use a correction for multiple testing, like a Bonferroni or something similar.

5) Methods
a) 510: typo in the p value threshold
b) Methods are thoroughly described
c) 572: The reference panel from which the UER LD scores came from should be mentioned here, for those unfamiliar with the default resources LDSC provides. Namely, which cohort did the LD scores come from?
d) 577: An r2 of 0.05 or greater seems liberal for combining loci. Is that a typo?
e) 600: Appreciate that you provide the code for gpu-coloc. Thanks for sharing that
f) The analysis code is not provided at the GitHub link shown. It looks like only some very simple table creation scripts are provided. Please provide the code used for the analyses presented in the manuscript.

6) Sup tables

a) Generally, they need more information. What is the meaning/interpretation of each column? What is the title of the table?
b) ST4: It’s unclear what this table is showing. There seem to be two different H4 probabilities shown for each row, which means two different things are being tested for colocalization with the metabolic trait. But what these two things are and why the table is structured this way is unclear.

(Remarks on code availability)
Point f under methods above listed also here
The analysis code is not provided at the GitHub link shown. It looks like only some very simple table creation scripts are provided. Please provide the code used for the analyses presented in the manuscript.

Referee #3

(Remarks to the Author)
I co-reviewed this manuscript with one of the reviewers who provided the listed reports.

(Remarks on code availability)
None of the actual analysis code is provided. What they have provided are some very simple scripts that reformat some tables. It is impossible to replicate any of their analyses or even see how they ran them.

For example, they provide a script called "disease_coloc.R", which one would reasonably assume is the code where they ran the "coloc" colocalization software. It is not. If you open the script up, you find a 35-line script that starts by loading the coloc results from some final results table that was previously created. Then the script does some minimal formatting and variable renaming. This is the case for every script in their provided GitHub repository.

In short, the code has not been made available to any extent.

Referee #4

(Remarks to the Author)
Request to referee the NMR section of the manuscript:
The NMR section does not present new experimental measurements but relies on well-established, thoroughly documented methodologies that have been developed and refined over the past 15 years. As no new NMR results are introduced in this manuscript, a full understanding of the approach and its context requires reference to earlier publications [73, 80, 81], where the foundational work is described in detail.

Given that the focus of the current manuscript lies outside the development of NMR methodology, and that it is not expected to be of direct technical interest to an NMR spectroscopist, this reliance on prior work appears entirely appropriate.

The data analysis, sample handling, and quality control as described in the earlier publcations have been conducted to a very high standard, adhering closely to best practices in the field. The methodology is applied with care and consistency, and is documented with sufficient transparency to ensure reproducibility. While no issues are anticipated, any potential problems that might arise in the future would be straightforward to identify and trace, thanks to the robustness and clarity of the procedures employed.

One additional remark: Even widely used abbreviations (e.g., MAF, QTL) should be spelled out upon first use to ensure clarity for all readers. Including a list of abbreviations at the end of the manuscript may also be helpful for reference.

Version 1:

Reviewer comments:

Referee #1

(Remarks to the Author)
The authors have effectively addressed all of my concerns; I have no further comments.

Referee #2

(Remarks to the Author)
The researchers present a revised manuscript that significantly expands their genetic colocalization analysis by incorporating data from major biobanks like UK Biobank and FinnGen, identifying over 932,000 events now accessible via a dedicated web browser. A key discovery highlights three colocalization events, suggesting plasma lactate may serve as a biomarker for platelet activation near specific genes. Furthermore, the team conducted extensive statistical fine-mapping of nearly 85,000 loci, pinpointing 3,000 distinct causal variants and revealing that low-frequency variants are generally easier to map due to less complex genetic linkage. Notably, these low-frequency variants were twice as likely to have clear functional impacts, such as missense or splice-altering effects, compared to common variants. Ultimately, the study emphasizes that integrating low-frequency variants into genome-wide association studies (GWAS) provides greater clarity

for identifying causal genes and their mechanisms of action.

While expanding colocalization efforts to include diseases beyond Type 2 DM and CAD is a positive step, I still find it hard to see these analyses providing any truly new biological insights. The two insights labeled as ‘novel’ relate to GlycA as an inflammation marker, which has been well established for over a decade (see PMID 32363327 review from 2014), and to plasma lactate, which may serve as a biomarker of platelet activation in early PE. The latter is entirely speculative, with no supporting evidence. The statement that these findings are backed “by observational studies linking elevated plasma lactate levels to increased mortality in patients with pulmonary embolism (ref 68–70)’ (line 293-95) may be confusing. These references discuss the link between lactate levels and prognosis after a pulmonary embolism, as well as the well-established connection between whole-body tissue hypoxemia and a switch to anaerobic metabolism caused by V/Q mismatch in the lungs and low cardiac output due to RV failure. Also, other clinical factors—mainly those related to Virchow’s triad—drive these events, many of which are complex or ‘environmental,’ such as hormone therapy, immobility, injury, and a generalized procoagulant state in cancers. Small changes in lactate production by platelets and other cells induced by these factors are unlikely to significantly contribute to platelet hyperactivity or thrombus formation, leading to DVT and PE. Additional questions include how to differentiate lactate originating specifically from platelets from that from other sources, and whether these findings could be examined using cis-MR analyses, similar to those involving genetic variants near genes that influence the GlycA signal (ORM1/2, HP, SERPINA1/3, TF). Could the authors perhaps consult a physician-scientist, such as a pulmonologist or hematologist, for further clinical insights into their findings (I may be mistaken, but I believe none of the authors are physicians)? Therefore, I feel the current presentation of these findings does not support the statement in the final paragraph of the discussion: “Our results demonstrate that even for well-studied metabolic traits, increasing GWAS sample size can still yield novel discoveries and biological insights,” and the authors might want to define novelty in this body of work based on some of their other findings.

Minor
Line 237: SpliceAI and AlphaGenome scores are provided. It would be helpful to include some context on how to interpret these scores. What does a score of 0.41 indicate? How can we determine that this number predicts disrupted splicing? What is the threshold for an effect?
Two figures are labeled as Figure S3.

In Figure S3 (GP6 locus), some of the metabolic traits are labeled with information that is difficult to interpret (e.g., I9_PLUMEMB_meta_out). Please use common names instead of abbreviations here.
Figure 3E: it doesn’t make sense to plot effect size, AlphaMissense prediction, and Pangolin splice loss score on the same scale. It’s better to only show the effect sizes, if anything.
NMR colocalization browser: it would be helpful to use full names rather than abbreviations for NMR traits. Many abbreviations are hard to interpret alone (e.g., total_p). The “closest gene” field seems to be case-sensitive. It would be helpful to clearly indicate this to users.

Referee #3

(Remarks to the Author)
I co-reviewed this manuscript with one of the reviewers who provided the listed reports.

(Remarks on code availability)
They have now uploaded what appears to be the actual analysis code, which addresses the issue from the original submission.

Open Access This Peer Review File is licensed under a Creative Commons Attribution 4.0 International License, which permits use, sharing, adaptation, distribution and reproduction in any medium or format, as long as you give appropriate credit to the original author(s) and the source, provide a link to the Creative Commons license, and indicate if changes were made.
In cases where reviewers are anonymous, credit should be given to 'Anonymous Referee' and the source.
The images or other third party material in this Peer Review File are included in the article's Creative Commons license, unless indicated otherwise in a credit line to the material. If material is not included in the article's Creative Commons license and your intended use is not permitted by statutory regulation or exceeds the permitted use, you will need to obtain permission directly from the copyright holder.
To view a copy of this license, visit https://creativecommons.org/licenses/by/4.0/

We would like to thank the reviewers for their constructive feedback and useful suggestions. In addition to many smaller updates, we have now made the following two major changes to the manuscript:

1. We have substantially expanded the genetic colocalisation analysis from only covering type 2 diabetes and coronary artery disease to all quantitative traits and disease endpoints from the UK Biobank, FinnGen and Million Veterans Program biobanks and their meta-analysis. We have also included new eQTL (INTERVAL) and pQTL (UKB-PPP) datasets to capture additional molecular mechanisms. We identified over 932,000 individual colocalisation events, which we now summarize in the revised colocalisation section of the manuscript. We have also set up a dedicated browser (https://elixir.ut.ee/eqtl/nmr-coloc) that makes it easy to query our colocalisation results based on the lead variant, closest gene, colocalising eQTLs and pQTL genes, and other criteria. In particular, we highlight three interesting colocalisation events between pulmonary embolism and lactate near three platelet-specific genes (GP6, GRK5, ZFPM2), suggesting that for these three genes, plasma lactate could act as a biomarker of platelet activation.

2. We have performed statistical fine mapping around all 84,762 genome-wide significant loci detected in the meta_EUR meta-analysis, identifying over 116,000 conditionally distinct signals, 27% of which were fine mapped to 3,000 distinct putative causal variants. We found that while 8.6% of the credible set lead variants were low-frequency (MAF < 1%), this proportion increased to 19.4% when focussing on confidently fine mapped (PIP > 0.8) variants, suggesting that, due to less extensive LD, low-frequency signals are easier to fine map than common signals. Importantly, these low-frequency (MAF < 1%) fine mapped variants were also twice as likely to be predicted as missense or splice-altering than common (MAF > 1%) fine mapped variants (23.1% vs 11.5%), thus providing a clear hypothesis about the causal gene and a likely mechanism of action. With association summary statistics for low-frequency variants now routinely available from large biobanks, our results highlight the added value of including low-frequency variants into GWAS discovery and interpretation.

Line numbers in this document refer to the **manuscript_annotated.pdf** file.

Referee expertise:

Referee #1: human genetics, QTL mapping

Referee #2: genetic epidemiology, cardiometabolic disease

Referee #4: metabolomics

Referees' comments:

Referee #1 (Remarks to the Author):

In this manuscript, Drs. Kaur Alasoo, Priit Palta, Ralf Tambets and colleagues present the world's largest genetic mapping of metabolites to date - outlining a massive-scale GWAS meta-analysis incorporating 249 circulating metabolites across up to 619,372 people in the Estonian Biobank and the UK Biobank. The authors integrate both common and less commonly-occurring (MAF<1%) variants into their analyses, identifying 88,604 genetic associations with metabolites - many of them novel (e.g., 27% novel associations for 3-Hydroxybutyrate and 85% novel associations for Lactate). The authors then used these metabolite QTLs (mQTLs) for downstream genetic colocalization analyses, focusing on coronary artery disease and type 2 diabetes - highlighting how inhibition of BCAA levels by modulating the BCAA catabolism pathway is not likely to reduce risk of type 2 diabetes. The authors make their results fully accessible via the GWAS Catalog and a dedicated interactive portal, providing the scientific community with a valuable resource for hypothesis generation and therapeutic target prioritization. Overall, the paper is very well written and the study's sample size is exceptional - affording the authors enough power to increase the discovery rate of its most recent comparator (Karjalainen et al., Nature, 2024) by approximately ten-fold. The authors' observations that leveraging high-quality imputations of rare variants can increase detection rates above and beyond those reported using rare variant burden testing via exome- or whole-genome analyses is also quite remarkable and will be of interest to the broader genetics community. Despite the manuscript's strengths, I have several suggestions for improvements before it should be considered for publication in Nature.

My major pieces of feedback for the manuscript are as follows:

1. In an attempt to differentiate this study from prior published metabolite QTL GWAS papers, the authors tested for associations with relatively rare genetic variants (i.e., those with minor allele counts <1%). However, they benchmarked their findings against a recent pre-print from Claudia Langenberg and team, which performed rare variant burden mapping of metabolic traits leveraging sequencing data readily available in UK Biobank. As such, the study's novelty mainly lies in the *scale* at which it is investigating rare variant associations - not necessarily the fact that it is investigating rare variant associations with metabolites in general. This should be clarified earlier in the manuscript.

We have now edited the Introduction to make it more explicit that while both Nag et al, 2023 and Zoodsma et al, 2025 studied the impact of rare and low-frequency variation on NMR metabolic traits with exome sequencing, neither study explored the full genome-wide spectrum of low-frequency genetic variation (page 2, lines 55-57):

“While some studies have combined rare genetic variants from exome sequencing with NMR metabolites (15, 16), the full genome-wide spectrum of low-frequency genetic variation has not been explored. ”

2. Furthermore - and perhaps more importantly - the value of this rare variant mapping for therapeutic target discovery or understanding of disease mechanisms is not entirely clear in this

iteration of the manuscript. The authors themselves acknowledge that rare mQTLs are difficult to implement as instruments for causal mapping (line 357: "…the BCKDK region lacks strong common variant associations and the low-frequency splice donor variant that we detected is too rare to have sufficient power for cis-MR"). Thus, what is the ultimate translational impact of rare variant mapping of metabolic traits? It appears - at least in this version of the manuscript - that its value is limited to greater understanding of basic biological mechanisms, but not necessarily a greater understanding of disease mechanisms or therapeutic targets.

In the revised version of the manuscript, we have now significantly expanded our coverage of low-frequency genetic variation by performing statistical fine mapping on all tested variants with MAF > 0.1% in the UKBB_EUR subset. In particular, we found that 8.6% of the fine mapped credible sets and 19.4% of the confidently fine mapped variants (posterior inclusion probability > 0.8) had MAF between 0.1% and 1%. Moreover, the low-frequency fine mapped variants (PIP > 0.8) were twice as likely to be predicted missense or splice-altering variants compared to common variants with MAF > 1% (23.1% vs 11.5%), suggesting that many of these low frequency variants can be easier to fine map and interpret relative to common variants. We now report this in the main text (page 8, lines 302-314):

> *"To further explore these low-frequency associations, we first focused on our fine mapping results. We identified 10,016 (8.6%) credible sets where the MAF of the variant with the highest PIP was between 0.1% and 1%. These credible sets belonged to 786 likely independent signal clusters and contained 583 distinct confidently fine mapped variants with (PIP > 0.8). Notably, 135/583 (23.1%) of the low-frequency fine mapped variants were predicted to be missense or splice variants (Table S5). In contrast, only 11.5% of the common (MAF > 1%) fine mapped variants were predicted to alter coding sequence or splicing, highlighting the increased interpretability of many low-frequency associations. Based on this analysis, we prioritised several low-frequency missense variants in known metabolic genes, such as variants in PCSK9, ABCA1 and ANGPTL4 associated with lipid traits, a missense variant (12-102840493-G-A) in phenylalanine hydroxylase (PAH) associated with phenylalanine and tyrosine, and four independent missense variants in the histidine ammonia lyase (HAL) gene associated with histidine (Table S5)."*

We have also substantially expanded our colocalisation analysis (see response to your point 3 below) and now report that ~2.5% of the colocalisations that we detect involve metabolic trait signals with MAF < 1% (page 7, lines 202-203):

> *"Finally, ~2.5% of these colocalisations involved low-frequency (MAF < 1%) metabolic trait loci."*

An important caveat here is that, with the exception of FinnGen and eQTL Catalogue, our colocalisation analysis relied on marginal summary statistics and was thus not able to detect colocalisation with many of the secondary fine mapped signals.

We now also explicitly discuss the potential of these low-frequency fine mapped signals for understanding disease mechanisms and prioritizing therapeutic targets in the Discussion (pages 16-17, lines 537-556):

> "*While we were still underpowered to detect many novel associations with rare variants (156 unique lead variants with MAF < 0.1%), our analysis clearly highlights the value of including low-frequency (MAF between 0.1% and 1%) variants into GWAS discovery and interpretation. While 8.6% of the credible sets had low-frequency lead variants, this proportion increased to 19.4% for confidently fine mapped (PIP > 0.8) variants, suggesting that, due to less extensive LD, low-frequency signals are easier to fine map than common signals. Importantly, these low-frequency fine mapped variants were also twice as likely to be predicted as missense or splice-altering than common fine mapped variants (23.1% vs 11.5%), thus providing a clear hypothesis about the causal gene and a likely mechanism of action. While traditional multi-cohort GWAS meta-analyses have been limited to common variants (1, 5), low-frequency variants are now routinely included in large biobanks such as FinnGen (28), MVP (29) and Pan-UKBB (27). As a result, 2,5% of our colocalisations involved metabolic trait loci with MAF < 1%. Furthermore, for many common complex diseases, cross-biobank meta-analyses (e.g. FinnGen+MVP+UKBB meta-analysis (6)) are now achieving comparable statistical power compared to traditional multi-cohort studies, thus making it possible to include low-frequency variants into colocalisation and cis-MR analyses to elucidate disease mechanisms and prioritise drug targets. However, taking full advantage of these low-frequency meta-analysis associations requires accurate fine mapping of conditionally distinct signals, which is currently an active area of research with several novel methods proposed (44, 89, 90).*"

3. Although the authors provide relatively interesting examples of how mQTLs can help us understand potential therapeutic mechanisms for Type 2 Diabetes (T2D) and coronary artery disease (CAD), I believe that they have missed an opportunity to present a more comprehensive mapping of causal associations between metabolites and complex illnesses by focusing on only two exemplary indications wherein metabolites likely play the broadest, most complex roles in disease pathophysiology. The authors themselves acknowledge that their GPU-accelerated re-implementation of the coloc algorithm is approximately 100-fold faster than the original R implementation; as such, I see no technical hurdles to conducting a more comprehensive mapping, which may reveal dozens of additional novel insights into disease mechanisms. The cornerstone exemplar in the current version of the paper - an assessment of the potential therapeutic effect of BCKDK inhibition on reducing T2D risk - is noteworthy but insufficient to fully illustrate the power of the mQTL dataset generated by the authors. Integrating genetic associations with a broader collection of complex, commonly occurring illnesses will strengthen the paper and increase its relevance for the broad readership of Nature.

Thank you for the good suggestion. In addition to T2D and CAD analyses presented in the initial version of the manuscript, we have now substantially expanded our colocalisation analysis to all available GWAS traits from three large biobanks (FinnGen, UK Biobank and Million Veterans

Program) and their meta-analysis (FinnGen-UKBB-MVP meta-analysis) as well as all molecular QTL data (pQTLs, eQTLs, sQTLs) available to us from the eQTL Catalogue, UKB-PPP and the INTERVAL cohort. This analysis allowed us to test for colocalisations at an unprecedented scale, identifying a total of 932,864 colocalisation events between NMR metabolic traits, molecular QTLs and phenome-wide GWAS traits (including hundreds of disease endpoints). These new results are presented in the main text and in the substantially revised Figure 2.

Pages 6-7, lines 189-215:

> *"To demonstrate how genetic associations with metabolic traits can help interpret disease associations, we used gpu-coloc (47) to colocalise all 86,886 signals from our meta_EUR analysis with GWAS summary statistics for up to 7,228 traits across three biobanks (FinnGen r12 (28), Pan-UKBB (27), Million Veterans Program (MVP) (29) and FinnGen+MVP+UKBB meta-analysis (6)) as well as gene expression, splicing and protein QTLs from the eQTL Catalogue (31), the INTERVAL eQTL study (32) and the UK Biobank Pharma Proteomics Project (UKB-PPP) (8). We also included GWAS meta-analysis summary statistics for coronary artery disease (CAD) from Aragam et al, 2022 (5) and for type 2 diabetes (T2D) from Suzuki et al, 2024 (1) (Figure 2B). Using a stringent colocalisation posterior probability threshold (PP.H4 > 0.9), we detected a total of 932,864 colocalisation events which involved all 249 studied metabolic traits at least once. We detected at least one colocalisation for 53.4% of the metabolic trait signals. Similarly, 20.2% of the CAD loci and 16.3% of the T2D loci colocalised with at least one metabolic trait. For biobanks, the proportion of colocalising loci ranged from 4.9% (Pan-UKBB) to 11% (MVP). The lowest rate of colocalisation was observed for eQTLs and pQTLs, where 1.5-4.2% of the loci colocalised with at least one metabolic trait. Finally, ~2.5% of these colocalisations involved low-frequency (MAF < 1%) metabolic trait loci.*
>
> *To better understand the patterns of shared colocalisations across complex traits, metabolic traits and molecular QTLs, we converted the 932,864 colocalisation events into a colocalisation graph, where independent association signals were defined as nodes and colocalisation events (PP.H4 > 0.9) between these nodes were defined as edges. This graph contained 2,946 connected components, which we defined as colocalisation clusters (see example in Figure S3). The sizes of the clusters ranged from 1 edge to 62,504 edges and the number of metabolic traits in each cluster ranged from 1 to 228. The two largest sources of colocalising associations were the Pan-UKBB (involved in 1,643 clusters) and the eQTL Catalogue (involved in 1,576 clusters), which likely reflects the very large number of associations present in those datasets (Figure 2B). These results can be explored in our interactive colocalisation browser available at https://elixir.ut.ee/eqtl/nmr-coloc."*

For your convenience, here is the revised version of Figure 2:

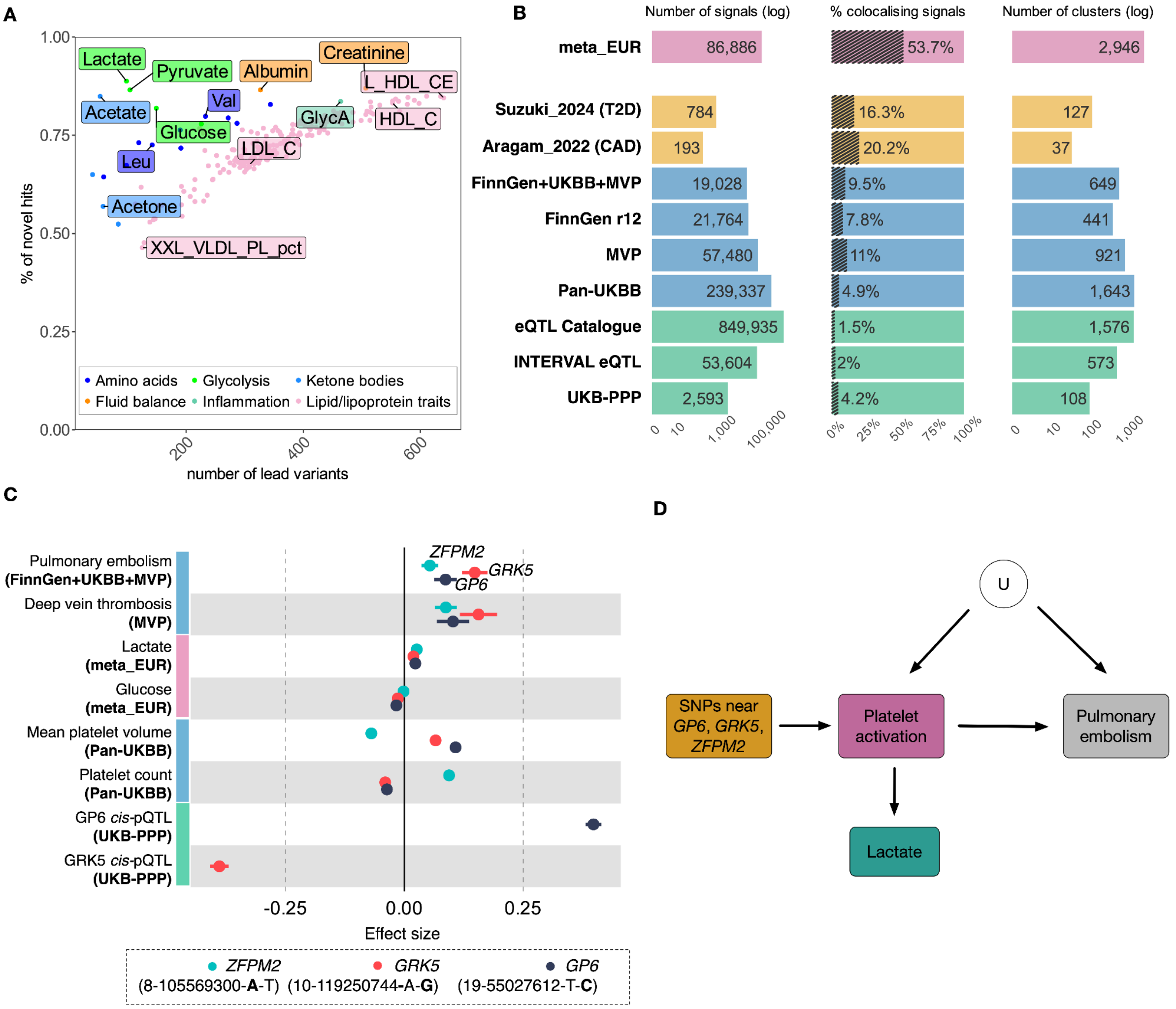

Figure 2. Known and novel genetic associations with metabolic traits. (**A**) Number of genome-wide significant loci ($p < 5\times10^{-8}$) detected for each metabolic trait and the proportion of those associations that were not detected by Karjalainen *et al*. (**B**) Overview of the datasets included in the colocalisation analysis. The first column shows the number of signals included for colocalisation from each dataset (log scale). The second column shows the proportion of these signals colocalising with at least one metabolic trait. The third column indicates the number of colocalisation clusters (log scale) involving at least one colocalisation from each dataset. (**C**) Forest plot of colocalising genetic associations at the *ZFPM2*, *GRK5*, and *GP6* loci between pulmonary embolism, deep vein thrombosis, lactate, glucose, mean platelet volume, platelet count and *cis*-pQTL signals for GP6 and GRK5 proteins. (**D**) Proposed causal model linking genetic variants at the *GP6*, *GRK5* and *ZFPM2* loci via platelet activation to increased pulmonary embolism risk. Lactate likely acts as a proxy readout of platelet activation without having a direct causal effect on pulmonary embolism risk. U - unmeasured confounders.

As you predicted, this analysis did reveal many additional novel insights into disease mechanisms. In the revised manuscript, we decided to focus on these three main results:

1. Recovering established associations with CAD and T2D (revised from initial version), page 7, lines 217-233)

"To assess the quality and scope of our resource, we focused on two common complex diseases with an established metabolic component: CAD and T2D. CAD was involved in 37 colocalisation clusters that contained a median of 177 metabolic traits (range 1-227). For example, a splicing QTL affecting the inclusion of exon 13 of 3-hydroxy-3-methylglutaryl-CoA reductase (HMGCR) colocalised with both CAD and LDL cholesterol as well as 127 other metabolic traits from our analysis (cluster 351, Figures S3-S4, Table S7). Similarly, a previously established and fine mapped liver-specific eQTL for SORT1 (1-109274968-G-T, PIP = 0.99) (49) colocalised with 182 metabolic traits (including LDL cholesterol) and CAD risk, as well as with a number of additional cardiovascular outcomes across the three independent biobank studies (cluster 61, Figure S5). In contrast, T2D was involved in 127 clusters that contained a median of 16 metabolic traits (range 1-228). As an example, a known pancreatic islet-specific enhancer variant 3-123346931-A-G (50) was detected as an eQTL in the PISA pancreatic islet dataset (51) for ADCY5 and colocalised with both glucose, T2D and other diabetes-related traits (cluster 207, Figure S5). Together, these examples highlight the utility of our meta-analyses results and demonstrate that large-scale colocalisation integrating metabolic and molecular traits enables the identification of tissue-specific regulatory mechanisms at known disease associated loci."

2. GlycA as a biomarker of chronic inflammation (page 8, lines 235-255)

"Next, we focused on the glycoprotein acetyls (GlycA) - a complex metabolic trait that has been linked to chronic inflammation (52, 53). There were 227 colocalisation clusters that involved GlycA as one of the metabolic traits. In FinnGen+UKBB+MVP meta-analysis, we detected at least one colocalisation with GlycA for 105 (out of 330) disease endpoints. The most common disease endpoints colocalising with GlycA were dyslipidemias (30 clusters), T2D (16 clusters), coronary atherosclerosis (15 clusters), hypertension (13 clusters) and gout (13 clusters), followed by many other diseases with fewer colocalisations. For example, a known variant in the IL6R gene (54) (1-154454494-A-C, rs2228145) was associated with GlycA in meta_EUR (p = 4.6x10-26) and colocalised with coronary atherosclerosis, ischemic heart disease, and several other cardiovascular endpoints (cluster 74). Furthermore, an established risk locus for inflammatory bowel disease (IBD) (55) (21-39094542-G-A, rs9808651) colocalised with GlycA and an eQTL for ETS2 transcription factor in stimulated monocytes (cluster 141). We next investigated if GlycA GWAS could provide added value over C-reactive protein (CRP) - another inflammatory biomarker for which large-scale GWAS studies exist (27, 56). We found that in 43/227 clusters, GlycA signal colocalised with CRP as profiled by Pan-UKBB. Similarly, the genetic correlation between GlycA and CRP in the UK Biobank was 0.53, which was much lower than the average of 0.96 for matched lab measurements from Pan-UKBB (Table S8, see Methods). These results indicate that GlycA and CRP capture complementary aspects of chronic inflammation, and suggest that our GlycA GWAS results can help to interpret a wide range of traits and diseases involving chronic inflammation."

3. Plasma lactate as a biomarker of platelet activation (pages 8-9, lines 257-298)

"To prioritise novel disease colocalisations while limiting the impact of horizontal pleiotropy that could complicate interpretation, we focused on the 291 colocalisation clusters that colocalised with at least one disease endpoint from the FinnGen+UKBB+MVP meta-analysis and involved five or less metabolic traits. This analysis revealed three high-confidence colocalisations between pulmonary embolism and plasma lactate at the GP6 (rs1654425, 19-55027612-T-C, cluster 65), GRK5 (rs10886430, 10-119250744-A-G, cluster 104) and ZFPM2 (rs6993770, 8-105569300-A-T, cluster 696) loci (Figure 2C). At all three loci, increased plasma lactate was associated with an increased risk of pulmonary embolism (FinnGen+UKBB+MVP meta-analysis) and an increased risk of deep vein thrombosis (MVP). For the glycoprotein VI platelet (GP6) and G protein-coupled receptor kinase 5 (GRK5) loci, we also detected colocalisations with the corresponding cis-pQTLs in the UKB-PPP plasma proteomics dataset, and for GRK5 we also detected a colocalising eQTL signal in platelets (57) as well as in whole blood (32). While we did not detect a colocalising cis-QTL effect at the zinc finger protein, FOG family member 2 (ZFPM2) locus, our lead variant (rs6993770) has previously been localised to a megakaryocyte-specific enhancer for other platelet traits (58). Finally, while all three loci also colocalised with both platelet count and mean platelet volume, the effect size directions were not consistent. At the GP6 and GRK5 loci, increased mean platelet volume and decreased platelet count were associated with increased risk for pulmonary embolism while at the ZFPM2 locus, both effects were the opposite (Figure 2C).

To further characterise these three loci, we focused on fine mapped credible sets from the GP6, GRK5 and ZFPM2 loci and performed colocalisation against all harmonized GWAS credible sets from the Open Targets Platform (59) using the colocalisation posterior probability (CLPP) method (see Methods) (47, 60). We found that the GP6 credible set (6 variants, max PIP = 0.48) colocalised (CLPP = 0.15) with a GWAS hit for platelet reactivity to collagen-related peptide (61), first reported in (62). Similarly, the fine mapped variant at the GRK5 locus (10-119250744-A-G, PIP = 0.99) colocalised (CLPP = 0.99) with a GWAS signal for thrombin-induced platelet activation in two independent studies (61, 63). This is consistent with experimental data in mice indicating that GRK5 is a negative regulator of platelet activation (64). The ZFPM2 fine mapped enhancer variant (8-105569300-A-T, PIP = 0.52) had pleiotropic effects on several blood cell type composition and cytokine traits (58, 65). Interestingly, at all three loci the alleles associated with increased lactate and increased pulmonary embolism risk were also associated with a decrease in plasma glucose (Figure 2C). Since activated platelets generate energy by converting glucose to lactate (66, 67), this suggests that at these three loci, plasma lactate might serve as a biomarker for platelet activation (Figure 2D) (13). This is also consistent with observational studies linking elevated plasma lactate levels to increased mortality in patients with pulmonary embolism (68–70). However, our analysis does not imply that plasma lactate itself has a direct causal effect on pulmonary embolism risk. In fact, when focussing on all genome-wide variants associated with

plasma lactate (including those not colocalising with pulmonary embolism), the association was inconclusive at best (Figure S6).”

4. The authors state that, "Our publicly available results will provide a valuable resource for GWAS interpretation and target prioritisation studies." I agree that this will be a valuable resource, and I commend the authors for already making the underlying summary statistics available via GWAS Catalog and a dedicated, interactive browser. This is a major strength of the paper. However, to fully optimize the utility of these data, the browser would benefit from three moderate updates:

○ First, when users search by gene (e.g., APOE), the query produces a table listing "top p-value in gene" and "phenotype". It omits the lead variant tagging each association, which is an important component to maintain for ease of interpretation.

Thank you for the suggestion. To clarify, we did not develop the GWAS browser available at https://nmrmeta.gi.ut.ee/ ourselves. Instead, we deployed the popular open source PheWeb software (https://github.com/statgen/pheweb) on our servers with our meta_EUR summary statistics loaded into it. The exact same web interface is used by several other biobanks and GWAS resources (see e.g. https://pheweb.org/, https://pheweb.jp/, https://metabolomics.baker.edu.au/, Million Veterans Program (https://phenomics.va.ornl.gov/web/cipher/pheweb)). However, we addressed this suggestion as well as we could. When searching by gene name, there is now one additional column in the results table listing the top variant. For example, this is how it looks for the *HMGCR* gene:

HMGCR

Phenotypes with the most-significant associations for this locus:

Top p-value in gene	Top variant	Phenotype
1.0e-300	5:75,360,714 T / C (rs12916)	Cholesteryl esters in medium VLDL
6.7e-297	5:75,360,714 T / C (rs12916)	Cholesterol in medium VLDL
7.9e-292	5:75,360,714 T / C (rs12916)	Concentration of IDL particles
5.8e-288	5:75,360,714 T / C (rs12916)	Phospholipids in IDL
6.0e-286	5:75,360,714 T / C (rs12916)	Concentration of large LDL particles
1.1e-283	5:75,360,714 T / C (rs12916)	Apolipoprotein B

Unfortunately, due to some internal design constraints of the PheWeb software, this field is sometimes empty:

GP6

Phenotypes with the most-significant associations for this locus:

Top p-value in gene	Top variant	Phenotype
7.9e-19	19:55,027,612 T / C (rs1654425)	Lactate
7.2e-12	19:55,027,612 T / C (rs1654425)	Glucose
3.7e-8	19:54,956,527 T / TA	Concentration of HDL particles
9.0e-8	19:54,956,527 T / TA	Total concentration of lipoprotein particles
2.7e-7		Apolipoprotein A1
3.0e-7		Concentration of medium HDL particles

To improve long term usability, we are also exploring options to migrate our browser to the newer PheWeb2 platform, which was just published a couple of days ago (https://www.nature.com/articles/s41588-025-02469-8).

○ Second, each phenotype contains an embedded hyperlink which seems as if it is meant to take the user to a new page after they click on it - either to the primary source of association (e.g., the paper in question) or to a new table showing the top variants and genes associated with that phenotype. Instead, it produces a LocusZoom Manhattan plot mapping hits at the general genetic locus in GWAS Catalog. This is not particularly intuitive, as after clicking on the phenotype, one would expect to either produce a full Manhattan plot showing all associations for that phenotype from chromosome 1 through 23, or alternatively to be taken to a new page listing all established genetic associations with that trait. Instead, clicking on the phenotype produces a LocusZoom which remains fixed at the original queried gene (e.g., APOE), which is confusing and counter-intuitive.

We agree that this behaviour can be somewhat counter-intuitive. However, many long-term users of the original PheWeb have gotten used to this behaviour and we feel that changing it in our browser could confuse them even more. Furthermore, the slightly modified version of PheWeb used by FinnGen (https://r12.finngen.fi/) and FinnGen+UKBB+MVP meta-analysis (https://public-mvp-ukbb.finngen.fi/) also have very similar behaviour.

○ Third, as an optional but highly valuable update, I would recommend integrating causal mapping results into the browser. This is especially relevant if the authors decide to expand beyond their initially limited focus on T2D and CAD.

Thank you for the suggestion. To make it easier to explore our expanded set colocalisation results (932,864 colocalisation events), we decided to set up a separate browser dedicated for displaying these results. The new NMR colocalisation browser is available at https://elixir.ut.ee/eqtl/nmr-coloc.

5. Approximately 3.3% (20,123) of the samples assayed in this study are from individuals of non-European ancestries. The authors should be commended for conducting ancestry-specific analyses in five non-European sub-cohorts of these 20,123 individuals - including people of African, Admixed American, Middle Eastern, East Asian and Central/South Asian ancestries.

However, the main text provides only a superficial overview of insights gleaned from these analyses, mainly providing a numeric breakdown of the total number of unique lead variants identified in each non-EUR cohort (with as few as 24 lead variants identified in Admixed Americans and as many as 143 lead variants identified in Africans). Were any mQTLs enriched in non-Europeans and/or depleted in Europeans? Was the median MAF enrichment of lead primary ancestry associations enriched for certain non-European ancestries (e.g., AFR) compared to others (e.g., CSA) when using the EUR cohorts as a benchmark? Are there any unique or interesting insights to be gleaned from these non-European participants? This section could benefit from further details.

We have now performed additional analysis where we quantified the proportion of non-EUR lead variants that were either not tested or rare (MAF < 0.1%) in the EUR subset of the UK Biobank. We report this in the main text (page 5, lines 165-167):

> *"Focussing separately on each genetic ancestry group, we found that between 9-48% of the lead variants had MAF < 0.1% in the UKBB_EUR analysis, with the highest proportion observed in the UKBB_AFR subset (68/143 lead variants) (Table S6)."*

Unfortunately our sample sizes in these non-EUR genetic ancestry groups were too small to draw any major conclusions. However, we now also refer to the study by Takeuchi et al, 2025 (https://doi.org/10.64898/2025.12.03.25341540) that profiled samples from 37,969 Japanese individuals on the same NMR platform.

We also included non-EUR summary statistics into our colocalisation analysis (all results available from Zenodo: https://zenodo.org/records/17945143), but due to the very small number of genome-wide significant loci in these ancestry groups, we did not detect any interesting novel colocalisations.

Other, minor comments on the manuscript are as follows:

6. The authors colocalized 87,373 signals from their meta-analysis of metabolic traits in Europeans with splicing QTLs and expression QTLs extracted from the eQTL catalog. Did the authors also consider integrating protein QTLs into this pipeline? If not, a short summary / justification would be helpful - perhaps this relates to the currently incomplete coverage of proteomic technologies in blood plasma (capturing only <50% of the circulating proteome). However, considering the controversies surrounding the use of eQTLs for fine-mapping of genetic associations with disease (discussed in greater detail by Mostafavi et al., Nature Genetics, 2023 https://www.nature.com/articles/s41588-023-01529-1), it is worth considering integrating of multiple QTL types beyond eQTLs.

Thank you for the suggestion. As we described in response to point 3 above, we have now substantially expanded our colocalisation analysis both with additional GWAS traits as well as molecular QTLs, including pQTL data from the UKB-PPP project and additional eQTL data from the INTERVAL cohort. We found that the additional pQTL data was certainly informative in a

number of cases (e.g. at the *GP6* and *GRK5* loci linking lactate to pulmonary embolism risk, Figure 2C).

7. The study leverages data generated using NMR metabolomics, primarily provided by Nightingale Health. While the NMR method offers high reproducibility, a standardized pipeline and the ability to scale to large biobank studies such as UKB and Estonia Biobank, it has limitations compared with other metabolic profiling techniques, such as mass spectrometry-based methods offered by companies like Metabolon. The latter technique can capture upwards of 4-5 times as many metabolites, with greater coverage of the major metabolic pathways as well as rarer metabolites, isomers and exogenous compounds. The limitations of NMR should be noted in the Discussion section.

We now briefly discuss mass spectrometry-based GWAS metabolic GWAS studies in the Discussion (page 17, lines 566-570):

> *"Lastly, our analysis was necessarily limited to the 249 (mostly lipoprotein) metabolic traits detectable by the Nightingale Health's NMR platform. Previous mass spectrometry-based metabolic trait GWAS studies have profiled a much broader spectrum of metabolites (up to 1,919 traits), but at the cost of significantly lower throughput (at most 19,994 individuals) (91–96)."*

8. The Discussion section ends somewhat abruptly by concluding an overview of the study's limitations. I suspect that this is due to limitations on word count; however, a sentence or two to conclude the manuscript would be appropriate to include at the end of the Discussion.

We have now added the following summary paragraph to the Discussion (page 17, line 585-589):

> *"Our results demonstrate that even for well-studied metabolic traits, increasing GWAS sample size can still yield novel discoveries and biological insights. However, as the discovery of associations increases, it is increasingly important to also invest in scalable tools and computational methods to support the interpretation and prioritisation of these associations."*

8. The manuscript is extremely well written overall but as is expected with any first draft, I noticed some minor grammatical or stylistic issues - for example:
○ Abstract: Consider changing "can be greatly improved by detailed understanding" to "can be improved by a gaining a greater understanding" or something similar.
We have now changed this.

9. Lines 36-27: "…can be greatly improved by detailed understanding of molecular consequences of genetic variation" - please include a, "the" before "molecular consequences".
Fixed.

10. Line 403: "…genetic variants from PPM1K, DBT and BCAT2 gene regions" - please add a "the" before "PPM1K".
Fixed.

Referee #2 (Remarks to the Author):

This study conducted a large-scale genome-wide association study (GWAS) meta-analysis of 249 circulating metabolic traits measured by nuclear magnetic resonance (NMR) spectroscopy, involving up to 619,372 individuals from the UK Biobank and Estonian Biobank. The analysis identified 88,604 associations between genetic loci and metabolic traits across 8,774 independent genomic regions. The authors performed GPU-facilitated systematic genetic colocalization analyses to explore shared genetic signals between metabolic traits, gene expression and splicing quantitative trait loci (QTLs), and the health outcomes of coronary artery disease (CAD) and type 2 diabetes (T2D). Additionally, cis-Mendelian randomization (MR) analyses were conducted to investigate potential causal relationships between metabolic traits and these complex diseases. The study highlighted extensive pleiotropy among metabolic traits, reinforcing previous findings, and provided insights into the biological pathways underlying these associations.

The main strength of this study compared to previous GWAS of metabolic traits is its significantly larger sample size (approximately 3-5 times larger than earlier studies and 40% larger than a parallel recent study described in an online preprint), enabling greater statistical power to detect novel genetic associations, including low-frequency variants. The inclusion of genetic associations with imputed X-chromosome variants also is a strength. Additionally, the integration of genetic colocalization and Mendelian randomization analyses facilitates deeper biological insights into the causal relationships between metabolic traits and complex diseases. The manuscript is also generally very well written and organized and the authors have produced a potentially impressive resource for the research community.

The study's weaknesses include limited genetic diversity, as most participants were of European ancestry, potentially limiting discovery and generalizability, but, more notably, limited novelty of biological insights described. The main problem in this respect is not the analytic approach, but rather the metabolomic platform which is biased and limited as well as dominated by blood lipid related traits and other metabolites that make up well established and well understood biological pathways studied for 50 years or more already (e.g. lipids, lipoprotein subclasses, fatty acid composition, inflammation markers, and various low-molecular metabolites like amino acids, ketones, and glycolysis-related metabolites). In this context, the newly identified genetic links within these well-established pathways is largely predictable as are the genetic correlation analyses showing pervasive pleiotropy.

We agree that this evaluation fairly reflects the initial version of our manuscript. In particular, the (somewhat) limited novelty of our results was also highlighted by Reviewer 1. While we agree that the range of metabolic traits captured by the NMR platform that we have used is certainly a limitation (and now explicitly acknowledge this in the Discussion, see response to point 7 from

Referee 1), we believe that our initial analysis was also limited by our too narrow focus on CAD and T2D as case studies. To overcome this, we have now substantially expanded our colocalisation analysis to all available GWAS traits from three large biobanks (FinnGen, UK Biobank and Million Veterans Program) and their meta-analysis (FinnGen-UKBB-MVP meta-analysis) as well as all molecular QTL data (pQTLs, eQTLs, sQTLs) available to us from the eQTL Catalogue, UKB-PPP and the INTERVAL cohort. This analysis allowed us to test for colocalisations at an unprecedented scale, identifying a total of 932,864 colocalisation events between NMR metabolic traits, molecular QTLs and phenome-wide GWAS traits (including hundreds of disease endpoints). These new results are presented in the main text and in the substantially revised Figure 2. See response to point 3 from Referee 1 for the detailed description of all of the changes in the current version of the manuscript.

In particular, we would like to highlight two novel insights that arose from our substantially expanded colocalisation analysis:

1. GlycA as a biomarker of chronic inflammation (page 8, line 235-255)

> *"Next, we focused on the glycoprotein acetyls (GlycA) - a complex metabolic trait that has been linked to chronic inflammation (52, 53). There were 227 colocalisation clusters that involved GlycA as one of the metabolic traits. In FinnGen+UKBB+MVP meta-analysis, we detected at least one colocalisation with GlycA for 105 (out of 330) disease endpoints. The most common disease endpoints colocalising with GlycA were dyslipidemias (30 clusters), T2D (16 clusters), coronary atherosclerosis (15 clusters), hypertension (13 clusters) and gout (13 clusters), followed by many other diseases with fewer colocalisations. For example, a known variant in the IL6R gene (54) (1-154454494-A-C, rs2228145) was associated with GlycA in meta_EUR (p = 4.6x10-26) and colocalised with coronary atherosclerosis, ischemic heart disease, and several other cardiovascular endpoints (cluster 74). Furthermore, an established risk locus for inflammatory bowel disease (IBD) (55) (21-39094542-G-A, rs9808651) colocalised with GlycA and an eQTL for ETS2 transcription factor in stimulated monocytes (cluster 141). We next investigated if GlycA GWAS could provide added value over C-reactive protein (CRP) - another inflammatory biomarker for which large-scale GWAS studies exist (27, 56). We found that in 43/227 clusters, GlycA signal colocalised with CRP as profiled by Pan-UKBB. Similarly, the genetic correlation between GlycA and CRP in the UK Biobank was 0.53, which was much lower than the average of 0.96 for matched lab measurements from Pan-UKBB (Table S8, see Methods). These results indicate that GlycA and CRP capture complementary aspects of chronic inflammation, and suggest that our GlycA GWAS results can help to interpret a wide range of traits and diseases involving chronic inflammation."*

2. Plasma lactate as a biomarker of platelet activation (pages 8-9, line 257-298)

> *"To prioritise novel disease colocalisations while limiting the impact of horizontal pleiotropy that could complicate interpretation, we focused on the 291 colocalisation clusters that colocalised with at least one disease endpoint from the FinnGen+UKBB+MVP meta-analysis and involved five or less metabolic traits. This*

analysis revealed three high-confidence colocalisations between pulmonary embolism and plasma lactate at the GP6 (rs1654425, 19-55027612-T-C, cluster 65), GRK5 (rs10886430, 10-119250744-A-G, cluster 104) and ZFPM2 (rs6993770, 8-105569300-A-T, cluster 696) loci (Figure 2C). At all three loci, increased plasma lactate was associated with an increased risk of pulmonary embolism (FinnGen+UKBB+MVP meta-analysis) and an increased risk of deep vein thrombosis (MVP). For the glycoprotein VI platelet (GP6) and G protein-coupled receptor kinase 5 (GRK5) loci, we also detected colocalisations with the corresponding cis-pQTLs in the UKB-PPP plasma proteomics dataset, and for GRK5 we also detected a colocalising eQTL signal in platelets (57) as well as in whole blood (32). While we did not detect a colocalising cis-QTL effect at the zinc finger protein, FOG family member 2 (ZFPM2) locus, our lead variant (rs6993770) has previously been localised to a megakaryocyte-specific enhancer for other platelet traits (58). Finally, while all three loci also colocalised with both platelet count and mean platelet volume, the effect size directions were not consistent. At the GP6 and GRK5 loci, increased mean platelet volume and decreased platelet count were associated with increased risk for pulmonary embolism while at the ZFPM2 locus, both effects were the opposite (Figure 2C).

To further characterise these three loci, we focused on fine mapped credible sets from the GP6, GRK5 and ZFPM2 loci and performed colocalisation against all harmonized GWAS credible sets from the Open Targets Platform (59) using the colocalisation posterior probability (CLPP) method (see Methods) (47, 60). We found that the GP6 credible set (6 variants, max PIP = 0.48) colocalised (CLPP = 0.15) with a GWAS hit for platelet reactivity to collagen-related peptide (61), first reported in (62). Similarly, the fine mapped variant at the GRK5 locus (10-119250744-A-G, PIP = 0.99) colocalised (CLPP = 0.99) with a GWAS signal for thrombin-induced platelet activation in two independent studies (61, 63). This is consistent with experimental data in mice indicating that GRK5 is a negative regulator of platelet activation (64). The ZFPM2 fine mapped enhancer variant (8-105569300-A-T, PIP = 0.52) had pleiotropic effects on several blood cell type composition and cytokine traits (58, 65). Interestingly, at all three loci the alleles associated with increased lactate and increased pulmonary embolism risk were also associated with a decrease in plasma glucose (Figure 2C). Since activated platelets generate energy by converting glucose to lactate (66, 67), this suggests that at these three loci, plasma lactate might serve as a biomarker for platelet activation (Figure 2D) (13). This is also consistent with observational studies linking elevated plasma lactate levels to increased mortality in patients with pulmonary embolism (68–70). However, our analysis does not imply that plasma lactate itself has a direct causal effect on pulmonary embolism risk. In fact, when focussing on all genome-wide variants associated with plasma lactate (including those not colocalising with pulmonary embolism), the association was inconclusive at best (Figure S6).”

Specific comments by section as well as suggestions for improvement are included below:

1) Abstract
a) Soften language: you predict that lowering BCAAs won't help T2D, didn't "find" it

We have now replaced "found" with "predict" in the Abstract.

2) Intro
a) 94: be consistent in "metabolite" vs. "metabolic trait". The latter is probably preferable, and it seems to be the term used more often in the manuscript.

We have now replaced all occurrences of "metabolite" with "metabolic trait".

3) Results
a) 125: What does "overlapping set of UKBB samples" mean? What the studies being overlapped with the UKB?

Our study and the study by Zoodsma *et al* are based on the same underlying UK Biobank raw NMR data generated by Nightingale Health.

b) 135: Great that you included the X chromosome in the analysis. That's so often excluded.

Thank you. The X chromosome was also excluded from the Genomics England imputation of the UK Biobank genotypes, which is why we used the TopMed imputation for the X chromosome and Genomics England imputation for the autosomes.

c) 156: Might be good to clarify what you mean specifically by "colocalized". Is it PP4 > 0.8? Do you include high PP3 probabilities too? Do you use a different threshold? I'm sure it's in the methods section, but it'd be helpful to mention briefly in passing here so readers know how to interpret these statements about colocalizations.

In response to request by Refree 1, we have now significantly revised and expanded the colocalisation analysis. In the updated description of the colocalisation results, we now also explicitly state the colocalisation threshold (page 7, line 195-196):

"Using a stringent colocalisation posterior probability threshold (PP.H4 > 0.9), …"

d) 185/186: how do you define "strongest metabolic association"? Based on P value from the GWAS? Coloc PP4 value for colocalization with the eQTL signal? Something else?

The original statement was based on the metabolic trait GWAS p-value, but in the updated colocalisation analysis we decided to remove the *LIPA* locus example from the main text (see also response to your next point) and replace it with a more general discussion of GlycA colocalisations with various disease endpoints. The updated text now reads (page 8, line 235-255):

“*Next, we focused on the glycoprotein acetyls (GlycA) - a complex metabolic trait that has been linked to chronic inflammation (52, 53). There were 227 colocalisation clusters that involved GlycA as one of the metabolic traits. In FinnGen+UKBB+MVP meta-analysis, we detected at least one colocalisation with GlycA for 105 (out of 330) disease endpoints. The most common disease endpoints colocalising with GlycA were dyslipidemias (30 clusters), T2D (16 clusters), coronary atherosclerosis (15 clusters), hypertension (13 clusters) and gout (13 clusters), followed by many other diseases with fewer colocalisations. For example, a known variant in the IL6R gene (54) (1-154454494-A-C, rs2228145) was associated with GlycA in meta_EUR (p = 4.6x10-26) and colocalised with coronary atherosclerosis, ischemic heart disease, and several other cardiovascular endpoints (cluster 74). Furthermore, an established risk locus for inflammatory bowel disease (IBD) (55) (21-39094542-G-A, rs9808651) colocalised with GlycA and an eQTL for ETS2 transcription factor in stimulated monocytes (cluster 141). We next investigated if GlycA GWAS could provide added value over C-reactive protein (CRP) - another inflammatory biomarker for which large-scale GWAS studies exist (27, 56). We found that in 43/227 clusters, GlycA signal colocalised with CRP as profiled by Pan-UKBB. Similarly, the genetic correlation between GlycA and CRP in the UK Biobank was 0.53, which was much lower than the average of 0.96 for matched lab measurements from Pan-UKBB (Table S8, see Methods). These results indicate that GlycA and CRP capture complementary aspects of chronic inflammation, and suggest that our GlycA GWAS results can help to interpret a wide range of traits and diseases involving chronic inflammation.*”

e) 190: I’m not sure I follow the logic here. The GWAS for CAD and GlycA were performed in different data sets. Plus, CAD is a binary outcome and GlycA is continuous. I’m not sure how the effect sizes here are directly comparable, nor how the effect estimate from the GlycA GWAS being smaller than the effect from the CAD GWAS implies the causal direction. Can you elaborate more on the logic here?

We have now removed this example from the main text and replaced it with a more general discussion of colocalisation with GlycA across a wide range of disease endpoints (see response to point d). We believe this new analysis reaffirms the role of GlycA as a generic marker of inflammation that is complementary to CRP.

Upon further consideration, we also agree that comparing standardised effect sizes across different traits with varying sources of variability and measurement noise is not necessarily meaningful. As a result, we now refrain from such comparisons in the main text.

f) 237+: This is a arguable the most novel finding here, highlighting the associations you found in other parts of the BCAA metabolic pathway by looking into rare variants.

Thank you.

g) 281: Just my personal preference, but I think Figure S6 would make sense as a main figure.

We did consider that, but ultimately decided to keep it as a supplementary figure. Part of the reasoning was that the heatmap of genetic correlations was only readable if we pre-selected a subset of metabolic traits and we did not want to impose such a selection in the main text.

4) Discussion

a) 409: This whole paragraph is focused on the Zoodsma preprint, pointing out ways the current study is superior to Zoodsma et al. This paragraph seems like maybe it would be appropriate in a message to an editor to justify this paper over the other, but in this manuscript, it just feels out of place and oddly critical of another lab's work. I'd prefer to see this paragraph removed.

We appreciate that the initial version of this paragraph might have seemed overly critical. Our intention was to highlight the primary differences between the two studies from the perspective of users who seek to use these summary statistics in their own follow-up analyses. We have now shortened this paragraph and have also more clearly highlighted aspects of the Zoodsma *et al* study that distinguish their analysis from ours (e.g. rare variant burden testing and exome-wide associations) (page 16, line 523-535):

> *"Our work is complementary to a parallel effort by Zoodsma et al that used an overlapping set of ~450,000 UK Biobank samples (15). First, including the Estonian Biobank into the meta-analysis increased the sample size by 40% and increased the number of genome-wide significant discoveries by approximately 65%. Secondly, using the latest set of imputed genotypes from both biobanks (37–39) allowed us to improve the coverage of low-frequency and rare variants. As a result, 8.6% of the fine mapped credible sets and 5.6% of the independent lead variants detected in our analysis had MAF of less than 1%. In contrast, Zoodsma et al. (15) performed additional rare variant burden testing and exome-wide association analysis, enabling us to replicate the imputation-based rare variant associations that we detected in the BCKDK and BCKDHA genes. Thus, our results illustrate how well-powered GWAS studies that test low-frequency and rare variants can help to reconcile the apparent differences in gene discovery from common-variant GWAS and rare variant burden testing studies (76, 88)."*

b) 436: They should at least use a correction for multiple testing, like a Bonferroni or something similar.

We have now revised our multiple testing strategy by applying the standard genome-wide significance threshold for common and low-frequency variants (MAF > 0.1%, ~15 million variants), but using a more stringent Bonferroni correction for the rare variants (MAF < 0.1%, up to 80 million variants). We now explain this in both in Results: (pages 3-4, lines 108-113):

> *"Based on minor allele frequency (MAF), we stratified these variants into three bins: common variants (MAF > 1%), low-frequency variants (MAF between 0.1% and 1%), and rare variants (MAF < 0.1%). For common and low-frequency variants, we used the*

standard genome-wide significance threshold of p < 5×10-8. For rare variants, we used a more stringent Bonferroni-corrected threshold of p < 6.25×10-10 (see Methods)."

and Methods (page 21, lines 715-720):

"For the common and low-frequency variants (MAF > 0.1%, ~15 million variants), we used the standard genome-wide significance threshold of p < 5×10-8. However, our analysis also included up to 80 million rare variants (MAF < 0.1%) for which the standard p-value threshold was too lenient. To be conservative, we treated all rare variant tests as independent and used the Bonferroni correction to establish a more stringent significance threshold of p < 0.05/80,000,000 = 6.25×10-10."

Furthermore, Table S3 now also contains a version Table 1 where we have used an additional Bonferroni correction step to further account for the number of metabolites tested. However, we note that it is a standard practice for large-scale GWAS resources (e.g. Pan-UKBB, https://www.nature.com/articles/s41588-025-02335-7) to report the number of associated loci using the standard $p < 5\times10^{-8}$ significance threshold even if the number of traits analysed is very large (e.g. 7,266 for Pan-UKBB).

5) Methods
a) 510: typo in the p value threshold
Fixed.

b) Methods are thoroughly described
c) 572: The reference panel from which the UER LD scores came from should be mentioned here, for those unfamiliar with the default resources LDSC provides. Namely, which cohort did the LD scores come from?

We have now added this information to the Methods (page 20, lines 705-709):

"Correlations were calculated between biobanks for each metabolic trait and between all metabolic traits in three of the largest datasets (EstBB, UKBB_EUR and meta_EUR) using the European reference panel LD scores from 1000 Genomes, as provided by the authors of LDSC (https://data.broadinstitute.org/alkesgroup/LDSCORE/eur_w_ld_chr.tar.bz2)."

d) 577: An r2 of 0.05 or greater seems liberal for combining loci. Is that a typo?

This is intentional. Our large sample size means that if the lead variant has a very small p-value then neighbouring variants with low LD ($r^2 < 0.5$ or $r^2 < 0.1$) could easily be significant. We have now clarified this in the Methods (page 21, lines 724-725):

"This was done to prevent very strong association signals (e.g. -log10p > 100) from 'bleeding' outside the 2 Mb window."

e) 600: Appreciate that you provide the code for gpu-coloc. Thanks for sharing that

Thank you.

f) The analysis code is not provided at the GitHub link shown. It looks like only some very simple table creation scripts are provided. Please provide the code used for the analyses presented in the manuscript.

Data analysis code has now been uploaded to https://github.com/ralf-tambets/EstBB-UKBB-metaanalysis .

6) Sup tables
a) Generally, they need more information. What is the meaning/interpretation of each column? What is the title of the table?
b) ST4: It's unclear what this table is showing. There seem to be two different H4 probabilities shown for each row, which means two different things are being tested for colocalization with the metabolic trait. But what these two things are and why the table is structured this way is unclear.

We have now added titles and explanations of the columns of the supplementary tables to the first sheet of the supplementary tables Excel file. Updated colocalisation results are now presented in supplementary table 7 and the names of columns have been explained on the first sheet of the Excel file.

Referee #2 (Remarks on code availability):

Point f under methods above listed also here
The analysis code is not provided at the GitHub link shown. It looks like only some very simple table creation scripts are provided. Please provide the code used for the analyses presented in the manuscript.

Data analysis code has now been uploaded to https://github.com/ralf-tambets/EstBB-UKBB-metaanalysis.
.

Referee #3 (Remarks to the Author):

I co-reviewed this manuscript with one of the reviewers who provided the listed reports.

Referee #3 (Remarks on code availability):

None of the actual analysis code is provided. What they have provided are some very simple scripts that reformat some tables. It is impossible to replicate any of their analyses or even see how they ran them.

For example, they provide a script called "disease_coloc.R", which one would reasonably assume is the code where they ran the "coloc" colocalization software. It is not. If you open the script up, you find a 35-line script that starts by loading the coloc results from some final results table that was previously created. Then the script does some minimal formatting and variable renaming. This is the case for every script in their provided GitHub repository.

In short, the code has not been made available to any extent.

We are sorry for omitting this in the previous version. We have now uploaded our data analysis code to GitHub:
https://github.com/ralf-tambets/EstBB-UKBB-metaanalysis.

Referee #4 (Remarks to the Author):

Request to referee the NMR section of the manuscript:
The NMR section does not present new experimental measurements but relies on well-established, thoroughly documented methodologies that have been developed and refined over the past 15 years. As no new NMR results are introduced in this manuscript, a full understanding of the approach and its context requires reference to earlier publications [73, 80, 81], where the foundational work is described in detail.

Given that the focus of the current manuscript lies outside the development of NMR methodology, and that it is not expected to be of direct technical interest to an NMR spectroscopist, this reliance on prior work appears entirely appropriate.

Thank you.

The data analysis, sample handling, and quality control as described in the earlier publcations have been conducted to a very high standard, adhering closely to best practices in the field. The methodology is applied with care and consistency, and is documented with sufficient transparency to ensure reproducibility. While no issues are anticipated, any potential problems that might arise in the future would be straightforward to identify and trace, thanks to the robustness and clarity of the procedures employed.

Thank you.

One additional remark: Even widely used abbreviations (e.g., MAF, QTL) should be spelled out upon first use to ensure clarity for all readers. Including a list of abbreviations at the end of the manuscript may also be helpful for reference.

We have now spelled out all abbreviations before their first use.

Referees' comments:

Referee #1 (Remarks to the Author):

The authors have effectively addressed all of my concerns; I have no further comments.

Referee #2 (Remarks to the Author):

The researchers present a revised manuscript that significantly expands their genetic colocalization analysis by incorporating data from major biobanks like UK Biobank and FinnGen, identifying over 932,000 events now accessible via a dedicated web browser. A key discovery highlights three colocalization events, suggesting plasma lactate may serve as a biomarker for platelet activation near specific genes. Furthermore, the team conducted extensive statistical fine-mapping of nearly 85,000 loci, pinpointing 3,000 distinct causal variants and revealing that low-frequency variants are generally easier to map due to less complex genetic linkage. Notably, these low-frequency variants were twice as likely to have clear functional impacts, such as missense or splice-altering effects, compared to common variants. Ultimately, the study emphasizes that integrating low-frequency variants into genome-wide association studies (GWAS) provides greater clarity for identifying causal genes and their mechanisms of action.

While expanding colocalization efforts to include diseases beyond Type 2 DM and CAD is a positive step, I still find it hard to see these analyses providing any truly new biological insights. The two insights labeled as 'novel' relate to GlycA as an inflammation marker, which has been well established for over a decade (see PMID 32363327 review from 2014), and to plasma lactate, which may serve as a biomarker of platelet activation in early PE. The latter is entirely speculative, with no supporting evidence. The statement that these findings are backed "by observational studies linking elevated plasma lactate levels to increased mortality in patients with pulmonary embolism (ref 68–70)' (line 293-95) may be confusing. These references discuss the link between lactate levels and prognosis after a pulmonary embolism, as well as the well-established connection between whole-body tissue hypoxemia and a switch to anaerobic metabolism caused by V/Q mismatch in the lungs and low cardiac output due to RV failure. Also, other clinical factors—mainly those related to Virchow's triad—drive these events, many of which are complex or 'environmental,' such as hormone therapy, immobility, injury, and a generalized procoagulant state in cancers. Small changes in lactate production by platelets and other cells induced by these factors are unlikely to significantly contribute to platelet hyperactivity or thrombus formation, leading to DVT and PE. Additional questions include how to differentiate lactate originating specifically from platelets from that from other sources, and whether these findings could be examined using cis-MR analyses, similar to those involving genetic variants near genes that influence the GlycA signal (ORM1/2, HP, SERPINA1/3, TF). Could the authors perhaps consult a physician-scientist, such as a pulmonologist or hematologist, for further clinical insights into their findings (I may be mistaken, but I believe none of the authors are physicians)? Therefore, I feel the current presentation of these findings does not support the statement in the final paragraph of the discussion: "Our results demonstrate that even for well-studied metabolic traits, increasing GWAS sample size can still yield novel discoveries and biological insights," and the authors might want to define novelty in this body of work based on some of their other findings.

Thank you for the detailed comments. We agree that the small changes in plasma lactate levels that we see are unlikely to have direct causal effect on thrombus formation and PE risk. We also explicitly state this in the manuscript: "However, our analysis does not imply that plasma lactate itself has a direct causal effect on pulmonary embolism risk. In fact, when

focussing on all genome-wide variants associated with plasma lactate (including those not colocalising with pulmonary embolism), the association was inconclusive at best (**Extended Data Fig. 3**)."

Our primary observation is that for the three genes with established biological roles in regulating platelet activation (*GRK5*, *GP6* and *ZFPM2*), there is a directionally concordant effect on both plasma lactate and PE risk, suggesting that plasma lactate can act as an (indirect) proxy readout for the effect of genetic variants on the protein function of these genes.

Minor
Line 237: SpliceAI and AlphaGenome scores are provided. It would be helpful to include some context on how to interpret these scores. What does a score of 0.41 indicate? How can we determine that this number predicts disrupted splicing? What is the threshold for an effect?
Both SpliceAI and AlphaGenome scores range from 0 to 1, with the vast majority of fine mapped credible set variants having scores close to 0. In our analysis, we used a threshold of 0.1 to identify potential splicing effects, which corresponded to top 3-3.8% of the fine mapped variants.

We have now added the following sentence to the Methods:
"Top 3% of the variants had a SpliceAI score > 0.1 and top 3.8% of the variants had AlphaGenome score > 0.1."

Two figures are labeled as Figure S3.
This has now been fixed.

In Figure S3 (GP6 locus), some of the metabolic traits are labeled with information that is difficult to interpret (e.g., I9_PLUMEMB_meta_out). Please use common names instead of abbreviations here.
The original Figure S3 is now Extended Data Figure 2. We have also re-labeled all traits with full names and have avoided confusing acronyms.

Figure 3E: it doesn't make sense to plot effect size, AlphaMissense prediction, and Pangolin splice loss score on the same scale. It's better to only show the effect sizes, if anything.

We have renamed Figure 3 to Figure 2. We agree that showing effect sizes and model predictions on the same scale was confusing. We have now split the original panel E into panels E and F. On panel E, we are now only showing the association effect sizes and their 95% confidence intervals while on panel F we now show the AlphaMissense and AlphaGenome scores.

NMR colocalization browser: it would be helpful to use full names rather than abbreviations for NMR traits. Many abbreviations are hard to interpret alone (e.g., total_p). The "closest gene" field seems to be case-sensitive. It would be helpful to clearly indicate this to users.

We have made two changes to colocalisation browser:
1. The 'closest gene' filter is no longer case-sensitive (e.g. filtering for grk5 and GRK5 will give the same results).
2. We have edited column descriptions on the About page (https://elixir.ut.ee/eqtl/about-nmr-coloc) and added a link to GitHub table that contains the full names of all tested metabolic traits:

“NMR trait — name of the NMR metabolic trait. Trait descriptions can be found (here)[https://github.com/ralf-tambets/EstBB-UKBB-metaanalysis/blob/main/data/biomarker_annotations.tsv).”
We also considered adding the full names to the colocalisation table, but this would have made the table too wide to display on most screens.

Referee #3 (Remarks to the Author):

I co-reviewed this manuscript with one of the reviewers who provided the listed reports.

Referee #3 (Remarks on code availability):

They have now uploaded what appears to be the actual analysis code, which addresses the issue from the original submission.
